# C_3_–C_4_ intermediacy in grasses: organelle enrichment and distribution, glycine decarboxylase expression, and the rise of C_2_ photosynthesis

**DOI:** 10.1093/jxb/erw150

**Published:** 2016-04-12

**Authors:** Roxana Khoshravesh, Corey R. Stinson, Matt Stata, Florian A. Busch, Rowan F. Sage, Martha Ludwig, Tammy L. Sage

**Affiliations:** ^1^Department of Ecology and Evolutionary Biology, University of Toronto, 25 Willcocks St., Ontario, ON M5S 3B2, Canada; ^2^Research School of Biology, Australian National University, Canberra, ACT 2601, Australia; ^3^School of Chemistry and Biochemistry, University of Western Australia, Crawley, WA 6009, Australia

**Keywords:** Arthropogoninae, bundle sheath, C_2_ Kranz anatomy, C_2_ photosynthesis, glycine decarboxylase, grasses, mitochondria, *Homolepis.*

## Abstract

Photorespiratory glycine shuttling and decarboxylation in bundle sheath (BS) cells exhibited by C_2_ species is proposed to be the evolutionary bridge to C_4_ photosynthesis in eudicots. To evaluate this in grasses, we compare anatomy, cellular localization of glycine decarboxylase (GDC), and photosynthetic physiology of a suspected C_2_ grass, *Homolepis aturensis*, with these traits in known C_2_ grasses, *Neurachne minor* and *Steinchisma hians*, and C_3_ *S*. *laxum* that is sister to *S*. *hians*. We also use publicly available genome and RNA-sequencing data to examine the evolution of GDC subunits and enhance our understanding of the evolution of BS-specific GDC expression in C_2_ and C_4_ grasses. Our results confirm the identity of *H*. *aturensis* as a C_2_ species; GDC is confined predominantly to the organelle-enriched BS cells in *H*. *aturensis* and *S*. *hians* and to mestome sheath cells of *N*. *minor*. Phylogenetic analyses and data obtained from immunodetection of the P-subunit of GDC are consistent with the hypothesis that the BS dominant levels of GDC in C_2_ and C_4_ species are due to changes in expression of a single GLDP gene in M and BS cells. All BS mitochondria and peroxisomes and most chloroplasts in *H*. *aturensis* and *S*. *hians* are situated centripetally in a pattern identical to C_2_ eudicots. In *S*. *laxum*, which has C_3_-like gas exchange patterns, mitochondria and peroxisomes are positioned centripetally as they are in *S*. *hians*. This subcellular phenotype, also present in eudicots, is posited to initiate a facilitation cascade leading to C_2_ and C_4_ photosynthesis.

## Highlight

C_2_ photosynthesis in grasses is facilitated by organelle enrichment in tandem with enhanced levels of GDC in the carbon-concentrating cells consistent with changes in expression of a single GLDP gene.

## Introduction

C_4_ photosynthesis has independently evolved >60 times in angiosperms (R.F. Sage *et al.*, 2011; Grass Phylogeny Working Group II, 2012). Within the angiosperms, the Poaceae represents the most prolific family of C_4_ origins, with approximately twice as many origins as any other family (Sage, 2016). C_4_ grasses also make up the greatest number of C_4_ species, comprising ~60% of the 8000 estimated number of C_4_ species (Still *et al.*, 2003; Sage, 2016). Roughly a quarter of global net primary productivity on land is due to C_4_ photosynthesis (Still *et al.*, 2003), of which the vast majority is contributed by grasses (Sage *et al.*, 1999). C_4_ grasses also have great significance for humanity as they dominate the fraction of biomass entering the human food chain as grain (maize, sorghum, and millets), sugar (sugarcane), and fodder for animals, and efforts are underway to engineer the C_4_ pathway into C_3_ grass crops such as rice and wheat to exploit the superior productivity of C_4_ photosynthesis (Peterhansel, 2011; von Caemmerer *et al.*, 2012). Given these considerations, there is now a great interest in understanding how C_4_ photosynthesis evolved in grasses, to understand both how this complex trait repeatedly arose, and how we might learn from the evolutionary examples to direct C_4_ engineering in major crops (Hibberd *et al.*, 2008).

Studies of C_4_ evolution are informed by the presence of species exhibiting intermediate stages between fully expressed C_3_ and C_4_ life forms within a single evolutionary clade (Sage *et al.*, 2014). Ideally, there should be C_3_–C_4_ relatives from multiple independent lineages of C_4_ photosynthesis to facilitate evaluation of evolutionary hypotheses using comparative approaches. Multiple independent clades provide the possibility to assess whether evolutionary trends are replicated, as they should be if C_4_ photosynthesis evolved along common trajectories (Heckmann *et al.*, 2013). To date, the majority (85%) of C_3_–C_4_ species occur in eudicots, with the genus *Flaveria* standing as the major group used in studies of C_4_ evolution (R.F. Sage *et al.*, 2011, 2014). *Flaveria* has over twice the number of intermediates (10) as any other evolutionary clade, and these form two distinct clades that each evolved C_3_- and C_4_-like species (McKown *et al.*, 2005; Lyu *et al.*, 2015). In addition to *Flaveria*, at least 11 other C_4_ evolutionary lineages have been identified with C_3_–C_4_ intermediates branching in sister positions to the C_4_ line (Sage *et al.*, 2014). Most of these have only one or two species, although in recent years there is evidence of three clades (*Heliotropium*, *Anticharis*, and *Blepharis*) potentially having more than five intermediates (Muhaidat *et al.*, 2011; Khoshravesh *et al.*, 2012; Fisher *et al.*, 2015). All but three of the C_4_ lineages with C_3_–C_4_ intermediates are eudicots. Among monocots, the genus *Neurachne* contains a C_3_–C_4_ intermediate that branches in a sister position to a C_4_ (Christin *et al.*, 2012). A recent study has reported C_3_, C_3_–C_4_, and C_4_ photosynthetic genotypes in *Alloteropsis semialata* (Lundgren *et al.*, 2015). The genus *Steinchisma* also contains C_3_–C_4_ intermediates (Brown *et al.*, 1983; Hylton *et al.*, 1988), but they lack close C_4_ relatives in their subtribe, Otachyrinae (Grass Phylogeny Working Group II, 2012). As a consequence of the discrepancy between C_3_–C_4_ numbers in eudicots and monocots, our understanding of C_4_ evolution is dominated by information from eudicot clades. If there is important variation in eudicot versus monocot patterns of C_4_ evolution, as suggested by recent theoretical treatments (Williams *et al.*, 2013), it could be missed because of low monocot representation in the C_3_–C_4_ intermediate population.

The South American subtribe Arthropogoninae is a hot-spot for C_4_ evolution within the grasses, with four putative distinct C_4_ origins, once in *Mesosetum*/*Arthropogon*, a second time in *Oncorachis*, and twice in *Coleataenia* (Grass Phylogeny Working Group II, 2012). As such, the Arthropogoninae is a strong candidate to contain numerous C_3_–C_4_ species. This possibility is bolstered by an image from *Homolepis aturensis* in Supplementary fig. S1 of Christin *et al.* (2013) that illustrates enlarged bundle sheath (BS) cells with chloroplasts arranged around the periphery in addition to chloroplast clusters adjacent to the vascular tissue. Although this centripetal chloroplast arrangement led to tentative identification of *H. aturensis* as C_4_ (Christin *et al.*, 2013), the presence of centrifugal chloroplasts in the BS cells is a common feature in C_3_–C_4_ intermediate species (Monson *et al.*, 1984; Sage *et al.*, 2014). Significantly, because the genus *Homolepis* is sister to a clade that contains only C_4_ species, it has been identified as a genus that might exhibit precursor traits that could have enabled the evolution of the C_4_ phenotype (Grass Phylogeny Working Group II, 2012). To evaluate this possibility, we have collected *H*. *aturensis* in Costa Rica for study.

A prominent physiological feature of C_3_–C_4_ intermediates is the transport of photorespiratory glycine from mesophyll (M) to BS cells for decarboxylation by glycine decarboxylase (GDC), with the released CO_2_ then being refixed by BS Rubisco (Monson and Rawsthorne, 2000). Photorespiratory glycine shuttling exhibited by C_3_–C_4_ intermediates has also been termed C_2_ photosynthesis in reference to the number of carbons shuttled from M to BS cells (Vogan *et al.*, 2007; Bauwe, 2011). The BS mitochondria of most C_2_ species examined to date contain the majority of the GDC within the leaf, with small amounts in M cells potentially for C1 metabolism (Hylton *et al.*, 1988; Morgan *et al.*, 1993; Rawsthorne *et al.*, 1998; Voznesenskaya *et al.*, 2001; Ueno *et al.*, 2003; Marshall *et al.*, 2007; Voznesenskaya *et al.*, 2010; Muhaidat *et al.*, 2011; T.L. Sage *et al.*, 2011). Decarboxylation of glycine in the BS cells establishes a glycine gradient between M and BS cells, and rapid movement to BS cells is facilitated by enhanced vein density in C_2_ relative to C_3_ species (Monson and Rawsthorne, 2000). Subsequent glycine decarboxylation within BS cells increases CO_2_ around BS Rubisco ~3-fold (Keerberg *et al.*, 2014), and the resulting increase in Rubisco efficiency reduces the CO_2_ compensation point in C_2_ species relative to C_3_ by 10–40 μmol mol^−1^ (Holaday *et al.*, 1984; Monson *et al.*, 1984; Vogan *et al.*, 2007; T.L. Sage *et al.*, 2011, 2013).

The functional GDC holoenzyme consists of four subunits encoded by individual genes, GLDH, GLDL, GLDP, and GLDT (Bauwe 2011). Decarboxylase activity of the complex is located in the P-subunit encoded by the GDLP gene. In *Flaveria*, the C_4_ photosynthetic mechanism was established through gradual pseudogenization of a ubiquitously expressed GLDP gene, and full activation of a second GLDP gene that shows BS-specific expression in C_3_ *Flaveria* species (Schulze *et al.*, 2013). The ancestral duplication of the GLDP gene in *Flaveria* is considered a genetic enabler of C_4_ evolution in the genus (Schulze *et al.*, 2013). BS cell-dominant expression of GDC has been reported in the C_2_ grass *Steinchisma hians* (=*Panicum milioides*; Hylton *et al.*, 1988). To date, the molecular evolution of this trait in *S*. *hians* or other C_2_ and C_4_ grasses has not been assessed. Schulze *et al.* (2013), highlighting the presence of two GLDP genes in rice [a member of the C_3_ BEP (Bambusoideae, Ehrhartoideae, Pooideae) clade] and a single copy in maize, sorghum, and *Setaria italica* [of the C_4_ PACMAD (Panicoideae, Arundinoideae, Chloridoideae, Micrairoideae, Aristoideae, Danthonioideae) clade], posited that the ubiquitously expressed GLDP gene(s) in C_4_ grasses were pseudogenized as in *Flaveria* and subsequently lost from the genomes.

The purpose of this study was to determine whether *H*. *aturensis* exhibits C_2_ photosynthesis or is a C_4_ species as previously suggested (Christin *et al.*, 2013). We compared anatomy, localization of GLDP, and photosynthetic physiology of *H. aturensis* with patterns previously identified in the C_2_ grasses *Steinchisma hians* and *Neurachne minor* (Morgan and Brown, 1979; Hylton *et al.*, 1988). Current phylogenies place the subtribe Otachyrinae, containing *Steinchisma*, as sister to the Arthropogoninae (Grass Phylogeny Working Group II, 2012). In addition, we examined *S. laxum* which has also been identified as a species that might provide information on the early stages of C_2_ and C_4_ evolution (Grass Phylogeny Working Group II, 2012; Sage *et al.*, 2013). Finally, we use genome sequence data from publicly available databases (the Phytozome and NCBI), as well as assembled RNA-sequencing (RNA-seq) from 16 additional grass species, to examine evolution of GLDP and genes encoding the other GDC subunits and provide a broader understanding of the evolution of BS-specific GDC expression in C_2_ and C_4_ grasses.

## Materials and methods

### Plant material

Plants of *Homolepis aturensis* Chase., *Steinchisma hians* Raf., and *S*. *laxum* (Sw.) Zuloaga obtained from sources described in Supplementary Table S1 at *JXB* online were grown at the University of Toronto in a greenhouse in 10–20 liter pots of a sandy-loam soil and were watered daily to avoid water stress. Fertilizer was supplied weekly as a 50:50 mixture of Miracle-Grow 24-10-10 All Purpose Plant Food and Miracle Grow Evergreen Food (30-10-20) at the recommended dosage (22ml of fertilizer salt per 6 liters; Scotts Miracle-Gro; www.scotts.ca). Plants of *Neurachne minor* from localities previously described (Christin *et al.*, 2012) were grown in a naturally illuminated glasshouse with mean temperatures of 25 °C/13 °C (day/night) at the Plant Growth Facility (PGF) of the University of Western Australia, Perth, Western Australia (latitude 33°89'S). To provide C_3_ and C_4_ grass species for comparison, we also examined leaves of PACMAD species *Dichanthelium oligosanthes* (Schult.) Gould (C_3_), *Panicum bisulcatum* Thunb. (C_3_), and two C_4_ species (*Panicum virgatum* L., NAD-ME subtype and *Setaria viridis* P. Beauv., NADP-ME subtype). Seed of these plants, obtained from sources described in Supplementary Table S1, were also grown at the University of Toronto.

### Leaf anatomy, ultrastructure, and immunolocalizations

The internal anatomy of leaves was assessed on sections sampled from the middle of the most recent, fully expanded leaves (one leaf per plant; three plants per species). Plants were sampled from 09:00h to 11:00h between April and August when day length was >11.5h and light intensity in the greenhouse regularly exceeded 1400 µmol photons m^−2^ s^−1^. The youngest cohort of fully expanded leaves was sampled in full sun for all procedures. Samples were prepared for light and transmission electron microscopy (TEM) to assess anatomy as previously described (T.L. Sage *et al.*, 2011, 2013; Stata *et al.*, 2014). For immunolocalization, tissue from the same region of the leaf was fixed overnight in 1% (v/v) paraformaldehyde and 1% (v/v) glutaraldehyde in 0.05M sodium cacodylate buffer. Tissue was then dehydrated and embedded in LR White (Voznesenskaya *et al.*, 2013). Immunolocalization of GLDP was conducted as outlined by Khosravesh *et al.* (2012). Primary and secondary antibody (18nm anti-rabbit IgG gold conjugate; Jackson Immunoresearch) dilutions were 1:50 and 1:20, respectively. Immunodetection of the Rubisco large subunit was modified from Ueno (1992). Sections were blocked in 0.5% BSA prior to incubation in primary antibody (1:100) for 3h. Incubation in secondary antibody (1:40; 18nm anti-rabbit IgG gold conjugate; Jackson Immunoresearch) was for 1h. To quantify all BS and M cellular features, TEM images from BS and M cells of the same grids used for immunogold labeling were analyzed using Image J software (Schneider *et al.*, 2012) as previously described (Sage *et al.*, 2013; Stata *et al.*, 2014). The anti-GLDP antiserum was commercially produced (GL Biochem) against a 17 amino acid peptide showing high conservation in both monocots and dicots. Antisera recognizing the Rubisco large subunit (RBCL) were obtained from AgriSera. Three replicate immunolocalizations were conducted on different days with each replicate including sectioned tissue from all species.

### Leaf gas exchange analysis

Gas exchange of intact, attached leaves was determined using a LiCor 6400 gas exchange system as previously described (Sage *et al.*, 2013). Measurement conditions were 31±1 °C and a vapor pressure difference between leaf and air of 2±0.2 kPa. For measurement of the response of net CO_2_ assimilation rate (*A*) to intercellular CO_2_ concentration (*C*_i_) at light saturation, leaves were first equilibrated to 1200–1500 µmol photons m^−1^ s^−1^ at an ambient CO_2_ concentration of 400 µmol m^−2^ s^−1^. Ambient CO_2_ levels were then reduced in steps to 30–50 µmol mol^−1^ (lower end of this range for C_2_ and C_4_ species, upper end of this range for C_3_ species), with measurements at each step after rate equilibration. The ambient CO_2_ was then returned to 400 µmol photons m^−1^ s^−1^, and *A* re-measured. If *A* was within 10% of the original rate, the CO_2_ concentration around the leaf was increased in steps to near 1600 µmol mol^−1^, with measurements made at each step. The linear initial slope of the *A*/*C*_i_ response was used as an estimate of carboxylation efficiency (CE).

For estimation of the apparent CO_2_ compensation point in the absence of day respiration (C_*_), the Laisk method was used as modified by Sage *et al.* (2013). Values of C_*_ were not estimated in C_4_ plants. Leaves were first equilibrated at 400 µmol mol^−1^ and a light intensity near saturation (900–1500 µmol photons m^−2^ s^−1^). The CO_2_ was then reduced to provide a *C*_i_ near 100 µmol mol^−1^, and, after stabilization, the rate was reduced in a series of steps to near the CO_2_ compensation point (Γ), with measurements at each step after signal stabilization. The *C*_i_ was then returned to near 100 µmol mol^−1^, and the procedure was repeated at a lower light intensity. This cycle was repeated such that when measurements were completed, there were 4–5 *A* versus *C*_i_ response curves with over five measurements at *C*_i_ values <100 µmol mol^−1^. Each *A* versus *C*_i_ response at a given light intensity was then fitted with a linear regression using the lowest 4–6 measurement points that fell on the regression. Points that fell below the regression line above ~80 µmol mol^−1^ were not included in the regression, as *A*/*C*_i_ responses below light saturation may be prone to non-linearity above 60–100 µmol CO_2_ mol^−1^ air. The estimate of C_*_ was taken as the *C*_i_ value where the 4–5 curves at different light intensities converged. Rarely, however, did all curves converge at the exact same *C*_i_; instead, they intersected with each other over a 5–10ppm range. In such cases, the middle of the intersects was taken as the C_*_ estimate. However, if there was evidence of a shift to lower photosynthetic photon flux density (PPFD) of the high light response, which often occurs in C_2_ species, the high light response was not included in the analysis (Sage *et al.*, 2013).

### Phylogenetic analysis of GDC subunit genes

Genome sequences for *Zea mays*, *Sorghum bicolor*, *Panicum hallii*, *Se. viridis*, *S. italica*, *Oryza sativa*, *Brachypodium distachyon*, and *B. stacei* were downloaded from the Phytozome v10.3 (https://phytozome.jgi.doe.gov/pz/portal.html). Polyploid species *Triticum aestivum* and *P. virgatum* were omitted. *Amborella trichopoda* was used as the outgroup, and we included the *Brassicaceae* species *Arabidopsis thaliana*, *Capsella grandiflora*, and *Boechera stricta* to show which duplications in the model plant *A. thaliana* are conserved across the land plants and which are lineage specific. RNA-seq data for 14 BEP clade species as well as the C_3_ PACMAD *Dicanthelium clandestinum* and its close C_4_ relative *Megathyrsus maximus* were downloaded from the NCBI short read archive (Supplementary Table S2). Reads mapping to each GDC subunit gene were identified using BLASTN with orthologs in *Z. mays* and *O. sativa* as queries (*S. bicolor* was used in lieu of *Z. mays* for one of the two GLDL paralogs as *Z. mays* appears to have lost this copy). For each gene, alignments were generated based on the Phytozome genomes using Muscle (Edgar, 2004), a highly conserved region was selected, and a consensus sequence was generated. These consensus sequences were used as reference for assembling sequences based on the retrieved reads using Geneious 8 (http://www.geneious.com).

The assemblies and sequences from Phytozome genomes were aligned using Muscle, trimmed using Trimal (Capella-Gutierrez *et al.*, 2009) with the ‘strict’ heuristic option, and used to generate Bayesian phylogenies using MrBayes (Ronquist and Huelsenbeck, 2003) as follows: four runs, four chains, GTR substitution model, 2 million generations for all trees except GLDT, which was run for 10 million generations to allow better convergence; ≥10 000 trees were sampled from portions of the end of each run where the average SD of split frequencies remained below 1%. Tree figures were generated using Fig Tree (http://tree.bio.ed.ac.uk/software/figtree).

### Data analysis

Results were analyzed with Sigmaplot version 12.5 (Systat Software., San Jose, CA, USA) using one-way ANOVA followed by a Tukey’s means comparison test. For characterization of leaf anatomy and ultrastructure, leaf samples were collected from three plants. For all traits measured, the values per plant were averaged to give one value for a plant. These individual plant values were the unit of replication for statistical analysis. For characterization of anatomical features, data from 3–5 sections per plant were averaged. For quantitative assessment of organelles in M and BS cells and GLDP immunodetection, the data from 10 imaged cells per cell type per plant were averaged. For leaf gas exchange, 4–15 measurements were conducted on 4–7 plants per species. In *Neurachne* species, vascular tissue is surrounded by two layers of cells, an outermost BS and innermost mestome sheath that functions in the C_4_ species as the site of CO_2_ refixation (Hattersley *et al.*, 1986). Comparing data collected from the mestome sheath of *N. minor* with data from the BS of the other grasses makes statistical comparisons invalid except for comparisons between either the presence or absence of GDLP in M versus the BS or mestome sheath cells. Hence, *N*. *minor* was not included in the statistical tests involving these other grasses.

## Results

### *Homolepis aturensis* possesses structural features common to C_2_ species

**Fig. 1.**
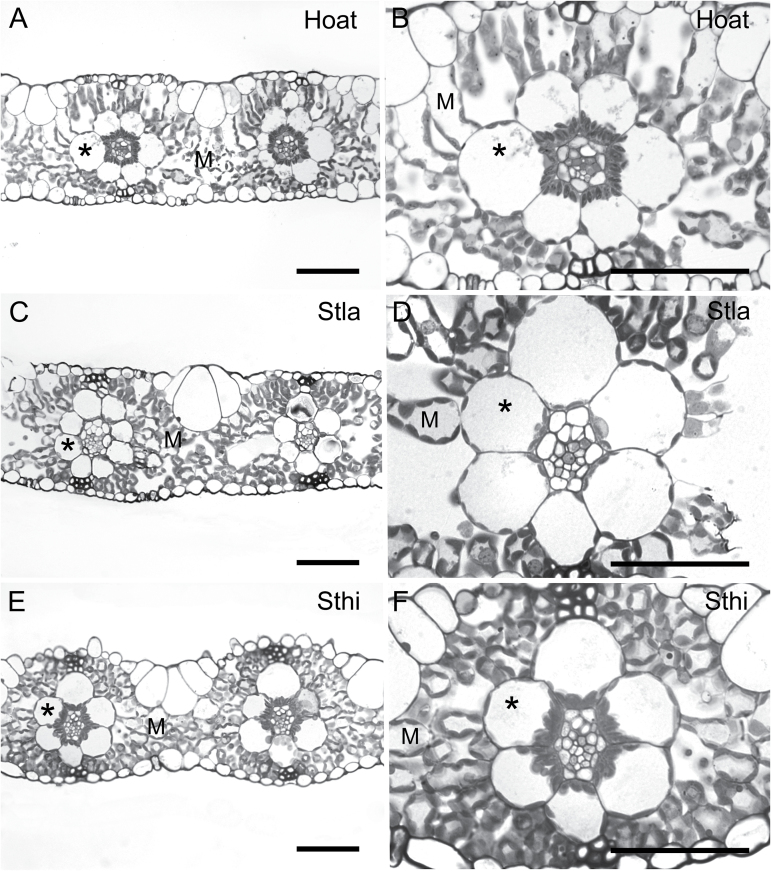
Leaf cross-sections from (A, B) *Homolepis aturensis* (Hoat), (C, D) *Steinchisma laxum* (Stla), and (E, F) *S. hians* (Sthi). M, mesophyll; asterisk, bundle sheath. Scale bars=50 µm.

Leaves of *H. aturensis* are anatomically similar to those of the C_2_ species *S*. *hians* and *S*. *laxum* with respect to M cell structure (Fig. 1). One layer of M cells extends from BS cells to the adaxial and abaxial epidermis (Fig. 1). Approximately six M cells separate the BS of adjacent veins in *S*. *laxum* whereas four cells separate the BS of adjacent veins in *H. aturensis* and *S*. *hians* (Fig. 1; Supplementary Table S3). The M:BS tissue ratio for *S*. *laxum*, *H*. *aturensis*, and *S*. *hians* is ~2 (Fig. 1; Supplementary Table S3), which is similar to that of the C_4_ species *P*. *virgatum* but >2.5 times less than that of the C_3_ species *D*. *oligosanthes* and *P*. *bisulcatum*. A large M and small BS volume contributes to an M:BS of almost 5 in *Se*. *viridis* even though there are only two cells between each vein (Supplementary Fig. S1C; Supplementary Table S3). M cells in *D*. *oligosanthes*, *P*. *bisulcatum*, and *Se*. *viridis* are more loosely packed and elongate than those of *P. virgatum* (Supplementary Fig. S1). The cellular features of *N*. *minor* are similar to those previously reported (Hattersley *et al.*, 1986), with 2–3M cells between veins (Supplementary Table S3).

**Fig. 2.**
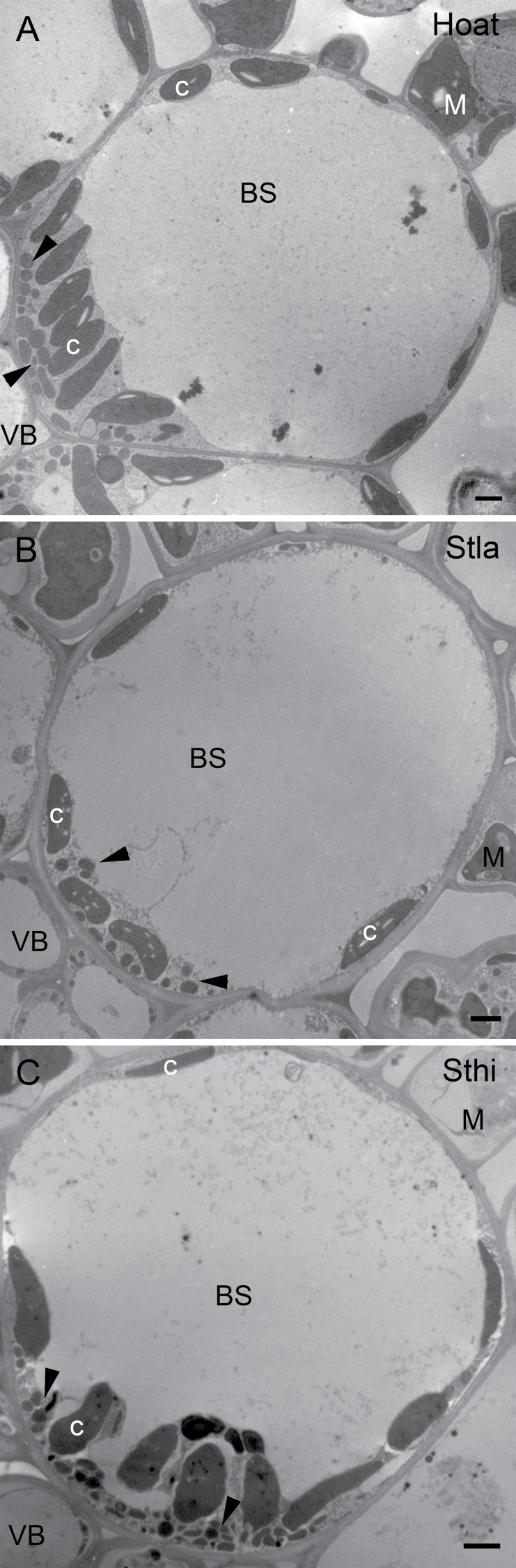
Bundle sheath ultrastructure of (A) *Homolepis aturensis* (Hato), (B) *Steinchisma laxum* (Stla), and (C) *S. hians* (Sthi). BS, bundle sheath; C, chloroplast; M, mesophyll; VB, vascular tissue; arrowheads, mitochondria. Scale bars=2 µm.

**Fig. 3.**
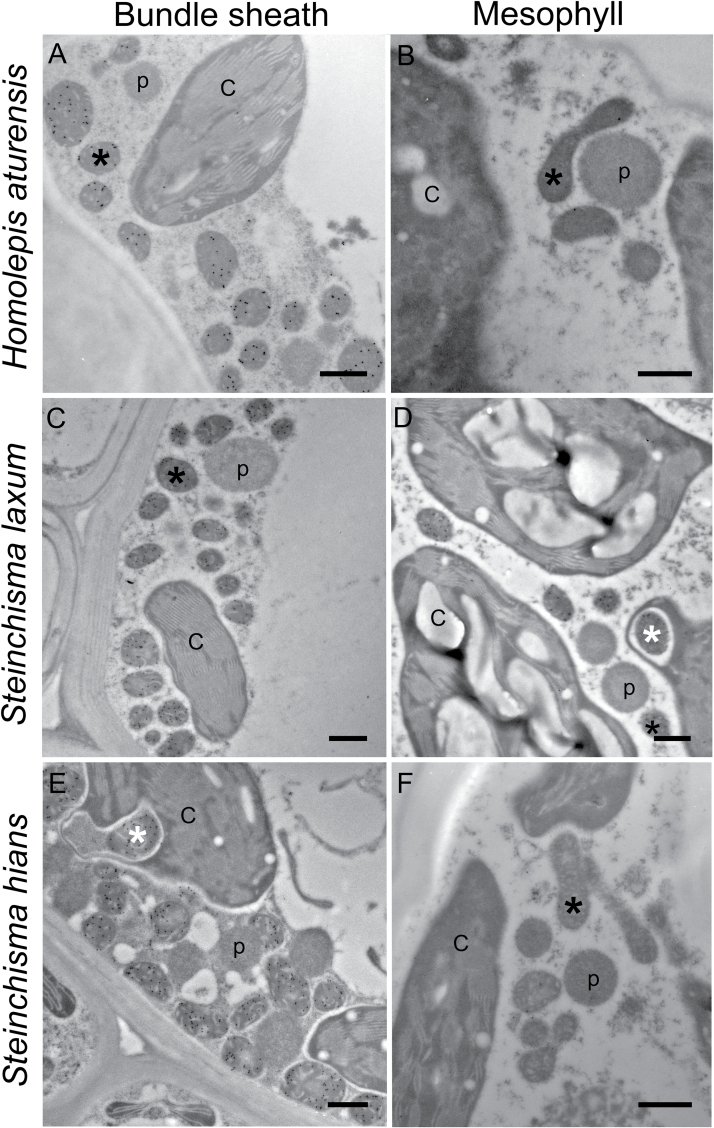
Immunolocalization of GLDP in bundle sheath and mesophyll cells of (A, B) *Homolepis aturensis*, (C, D) *Steinchisma laxum*, and (E, F) *S. hians*. C, chloroplasts; p, peroxisomes; black asterisk, mitochondria; white asterisk, mitochondria surrounded by chloroplast. Scale bars=500nm.

The BS cells of *H. aturensis* contain chloroplasts arranged around the periphery, with a significantly greater number clustered centripetally (Figs 1A, B, 2A; Supplementary Table S4). Mitochondria and peroxisomes localize almost exclusively to the centripetal BS pole (Fig. 2A; Supplementary Table S4). These spatial arrays of chloroplasts, mitochondria, and peroxisomes are similar in BS cells of *S*. *hians* (Figs 1E, F, 2C; Supplementary Table S4). As observed for *H*. *aturensis* and *S*. *hians*, a significantly greater number of mitochondria and peroxisomes are positioned at the centripetal BS pole in *S*. *laxum*, although chloroplasts are arranged equally around BS cells (Fig 1C, D; Supplementary Table S4). BS mitochondria are commonly surrounded by chloroplasts in *S*. *hians* (Fig 3E), *H*. *aturensis*, and *S*. *laxum* in a pattern similar to previous reports for *Steinchisma* species (Brown *et al.*, 1983). In contrast to centripetal chloroplasts displayed with their long axis parallel to the BS wall in *S*. *laxum*, the long axis of these chloroplasts in *H*. *aturensis* and *S*. *hians* are perpendicular to the BS wall (Figs 1, 2). BS chloroplasts are situated primarily in a centrifugal position in C_3_ grasses *D*. *oligosanthes* and *P. bisulcatum* (Supplementary Table S4; Supplementary Figs S1, S2), as noted in C_3_ eudicots (Muhaidat *et al.*, 2011; Sage *et al.*, 2013). Approximately 65–69% of mitochondria and 19% (*D*. *oligosanthes*) and 41% (*P*. *bisulctum*) of peroxisomes, respectively, are located in the centripetal position of the C_3_ grasses (Supplementary Table S4).

Quantitative parameters of organelle traits of BS and M cells are summarized in Supplementary Table S5. BS organelle parameters of *H*. *aturensis* and *S*. *hians* are not statistically different from each other except for a significantly greater number of mitochondria per planar cell area in *S*. *hians*. Mitochondria planar area per planar BS cell area of *H*. *aturensis* and *S*. *hians* is significantly greater than for *S*. *laxum*. In addition, mitochondria number per planar BS cell area, chloroplast and peroxisome number, and planar area per planar BS cell area are significantly greater in *S*. *hians* than in *S*. *laxum*. Results from a one-way ANOVA on ranks comparing peroxisome planar area per planar BS cell area between *S*. *hians*, *S*. *laxum*, and *H*. *aturensis* only indicated that this trait was significantly higher in *S*. *hians* and *H*. *aturensis* than in *S*. *laxum* (*P*≤0.001). The C_3_ grasses have the lowest mitochondria planar area per planar BS cell area; however, BS mitochondria parameters of C_4_ grasses are not different from those of the C_2_ grasses *H*. *aturensis* and *S*. *hians*. Mitochondria, peroxisomes, and chloroplasts are abundant in mestome sheath cells of *N*. *minor* (Hattersley *et al.*, 1986; Sage *et al.*, 2014). Unlike *H*. *aturensis*, *S*. *hians*, and *S*. *laxum*, organelles are not polarized in their distribution (Supplementary Fig. S3), but are instead positioned around the mestome sheath cell periphery (Hattersley *et al.*, 1986; Sage *et al.*, 2014).

When considering M cell organelle features, significantly fewer mitochondria and peroxisomes are observed in C_4_ species relative to the C_3_ species and the C_2_ species *H*. *aturensis*, *S*. *hians*, and *S*. *laxum* (Supplementary Table S5). The C_4_ species as well as *H*. *aturensis* have significantly lower chloroplast planar area per M planar cell area relative to *S*. *hians*, *S*. *laxum*, and the C_3_ grass species, and these changes result from either smaller (*H*. *aturensis*, *P*. *virgatum*) or fewer (*Se*. *viridis*) M cell chloroplasts (Supplementary Table S5). M cells of C_4_-like and C_4_ *Flaveria* and other C_4_ species have recently been reported to have significant reductions in chloroplast volume (Stata *et al.*, 2014, 2016).

### *Homolepis aturensis* exhibits C_2_ levels of GLDP in bundle sheath and mesophyll cells

Results from quantification of gold particles conjugated to secondary antibodies that bind to anti-GLDP are summarized in Supplementary Table S5. GLDP is almost exclusively located in BS cells of *H*. *aturensis* and *S*. *hians*, and mestome sheath cells of *N*. *minor* (Fig. 3A, B, E, F; Supplementary Fig. S3). Both BS and M cells contained high levels of GLDP labeling in *S*. *laxum* (Fig. 3C, D). In comparison with all other C_3_ species examined, *S*. *laxum* had the highest GLDP labeling in BS mitochondria (Supplementary Table S5). These patterns in GLDP distribution in M and BS mitochondria of *S*. *laxum* and *S*. *hians* are similar to earlier reports on these species (Hylton *et al.*, 1988). The gold density in C_3_ species *D*. *oligosanthes* and *P*. *bisulcatum* is higher in mitochondria of M than BS cells and is significantly greater on a planar M cell area basis than in those of *H*. *aturensis*, *S*. *hians*, and the C_4_ species (Supplementary Table S5; Supplementary Fig. S4).

### *Homolepis aturensis* exhibits C_2_ levels of Rubisco in bundle sheath and mesophyll cells

Rubisco occurs in both M and BS cell chloroplasts of *H*. *aturensis*, and this pattern was similar to that observed in *S*. *hians*, *S*. *laxum*, and M and mestome sheath cells of *N*. *minor* (Supplementary Fig. S5). As expected, Rubisco labeling is present only in the BS chloroplasts of the C_4_ species (Supplementary Fig. S6). Although Rubisco is also present in the M and BS cells of the C_3_ species *D*. *oligosanthes* and *P*. *bisulcatum*, there is qualitatively less labeling in the BS cells (Supplementary Fig. S6).

### *Homolepis aturensis* exhibits photosynthetic characteristics of a C_2_ species

**Fig. 4.**
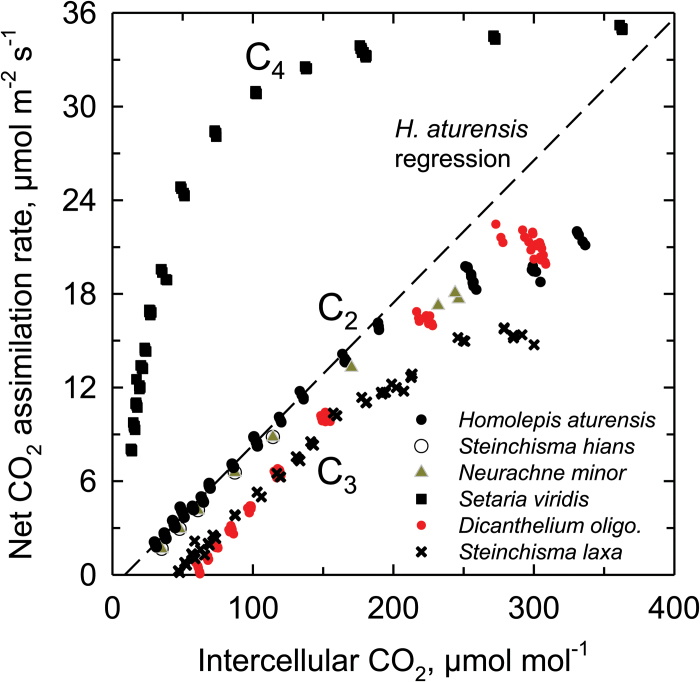
Response of net CO_2_ assimilation rate to intercellular CO_2_ concentration in *Homolepis aturensis*, two C_2_ species (*Neurachne minor* and *Steinchisma hians*), a C_4_ species (*Setaria viridis*), a C_3_ species (*Dicanthelium oligosanthes*), and a C_3_ species with proto-Kranz anatomy (*Steinchisma laxum*). Measurement conditions were 31±1 °C and saturating light intensities (1200–1500 µmol m^−2^ s^−1^). The curves shown are representative of 2–3 individual *A/C*_i_ responses per species. The linear regression for points below 100 µmol mol^−1^ is shown for *H. aturensis* (dashed line).

Values of *A* at 400 µmol CO_2_ mol^−1^ air are statistically similar, being 21±3 (mean±range) µmol m^−2^ s^−1^ for all species in the study except for the C_4_ plant *Se. viridis*, which has higher *A* (Table 1). Thus, any differences in carboxylation efficiency (CE), Γ, or intrinsic water use efficiency (estimated as *A/g*_s_ at 400 µmol CO_2_ mol^−1^ air) should reflect photosynthetic pathway effects and not variation in photosynthetic capacity. Comparison of the *A* versus *C*_i_ responses at saturating light intensities show three equivalent sets of curves that corresponded to the photosynthetic pathway (Fig. 4). C_3_ and C_2_ species have similar responses, with the exception that Γ was reduced ≥30 µmol mol^−1^ in the C_2_ species; consequently, their *A*/*C*_i_ responses were shifted to lower *C*_*i*_ values. *Homolepis aturensis* has *A*/*C*_i_ responses identical to those of *S. hians* and *N. minor*, leading us to classify *H. aturensis* as a C_2_ species. Carboxylation efficiencies and *A*/*g*_*s*_ of all the C_2_ and C_3_ species are statistically identical and less than those of the C_4_ species (Table 1).

**Table 1.** Summaries of gas exchange values for species included in this study. Values are means ±SE.

	**Species**	***n***	C_*_ µmol mol^−1^	**CE** mol m^−2^ s^−1^	***A***_**at 400**_ µmol m^−2^ s^−1^	***A/g***_***s* at 400**_ µmol mol^−1^
**C**_**3**_ **species**						
	*Dicanthelium oligosanthes*	3, 6	48±1 a	0.13±0.01 b	24.5±1.5 b	56±2
	*Panicum bisulcatum*	6, 6	50±2 a	0.11±0.01 b	18.7±1.7 b	62±8
**C**_**3**_ **-Protokranz**						
	*Steinchisma laxum*	5, 7	53±1 a	0.12±0.0 b	21.7±2.4 b	62±11
**C**_**2**_ **species**						
	*Homolepis aturensis*	4, 7	10.8±1.4 c	0.09±0.01 b	19.8±1.4 b	57±10
	*Neurachne minor*	3, 3	20.0±2.5 b	0.11±0.02 b	17.7±1.9 b	43±4
	*Steinchisma hians*	5, 8	11.8±1.4 c	0.10±0.01 b	21.6±1.1 b	58±7
**C**_**4**_ **species**						
	*Panicum virgatum*	0, 2	NA	0.36±0.01 a	24.5±2.5 b	152±5
	*Setaria viridis*	0, 3	NA	0.54±0.05 a	31.9±0.8 a	76±12

Measurement temperature was 31±1 °C.

Sample sizes given are *n* = 3-5 for C_*_ estimates, and *n* = 3–8 for all other data, with the exception of *P*. *virgatum* where *n*= 2).

Letters indicate statistical groupings at *P*<0.5 via one-way ANOVA followed by a Student’s–Neumann–Kuehls test.

NA, not applicable.

400 refers to ambient CO_2_ concentration in µmol mol^−1^.

**Fig. 5.**
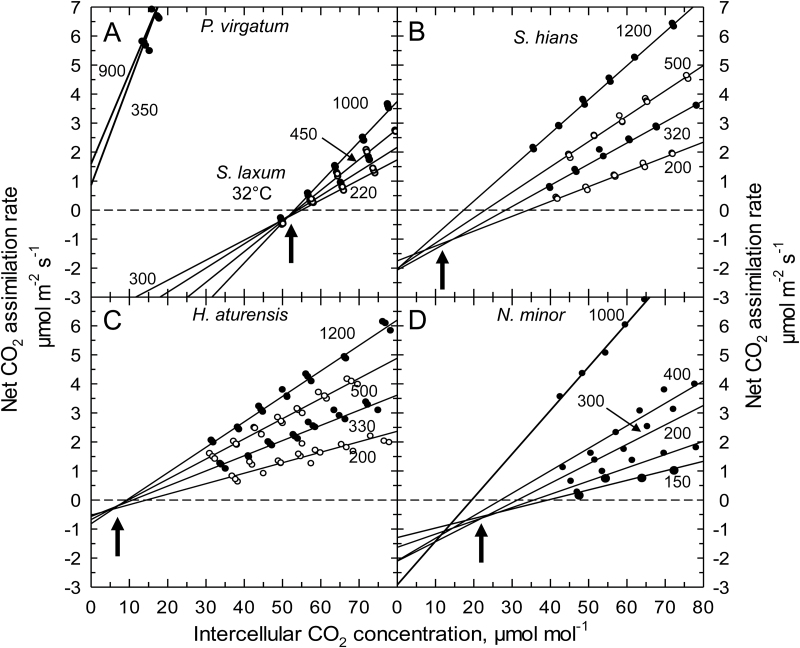
Representative *A/C*_i_ responses below 80 µmol mol^−1^ determined on single leaves at four distinct light intensities of (A) *Panicum virgatum* (C_4_) and *Steinchisma laxum* (C_3_ proto-Kranz), (B) the C_2_ species *Steinchisma hians*, (C) the C_2_ species *Homolepis aturensis*, and (D) the C_2_ species *Neurachne minor*. Measurement light intensities are indicated beside each curve in µmol photons m^−1^ s^−1^. The C_*_ estimate is indicated by arrows. Curves shown are representative of 3–6 measurement sets per species, except for *P. virgatum* where two sets of measurements were obtained. Measurement temperature was 31±1 °C.

**Fig. 6.**
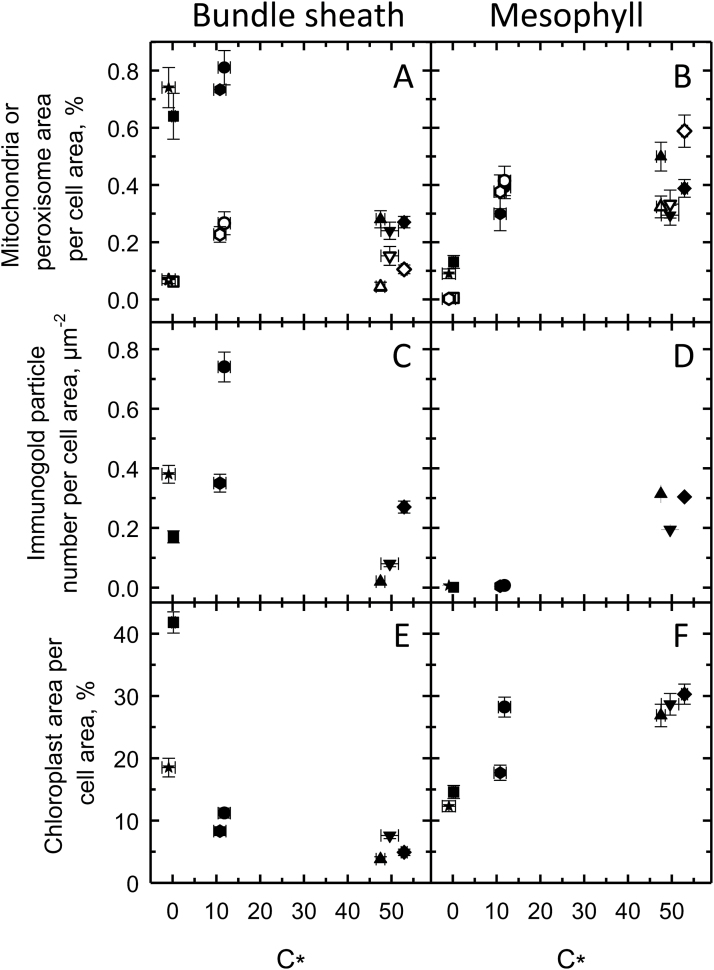
C_*_ (Γ, for C_4_ species), organelle traits, and GLDP labeling in bundle sheath (A, C, E) and mesophyll (B, D, F) cells of *Dichanthelium oligosanthes* (triangle, C_3_), *Panicum bisulcatum* (inverted triangle, C_3_), *Steinchisma laxum* (diamond, C_3_), *S. hians* (circle, C_2_), *Homolepis aturensis* (hexagon, C_2_), *Se. viridis* (square, C_4_), and *P. virgatum* (star, C_4_). Filled and open symbols represent mitochondria and peroxisomes, respectively. Mean ±SE.

In the C_3_ species *D*. *oligosanthes* and *P*. *bisulcatum*, we observed C_*_ values near 50 µmol mol^−1^ (Table 1) which is typical for C_3_ species at 31 °C (Busch *et al.*, 2013). *Steinchisma laxum* had a similar C_*_ value (53 µmol mol^−1^; Table 1; Fig. 5 A) indicating it is functionally C_3_ despite the potential function of the BS organelles. The C_*_ values of the C_2_ species were 10.8−20 µmol mol^−1^ (Table 1; Fig. 5B–D); the 10.8 value is on the lower end of C_*_ for species with this physiology (Edwards and Ku, 1987). In the grasses studied here, lower C_*_ values correspond to higher values of BS mitochondria per planar cell area (Fig. 6A) and higher BS GLDP density per planar cell area, except for *Se*. *viridis* (Fig. 6C). C_*_ was lower in species where BS chloroplast area per planar cell area was greater and M chloroplast area per planar cell area was lower (Fig. 6E, F).

**Fig. 7.**
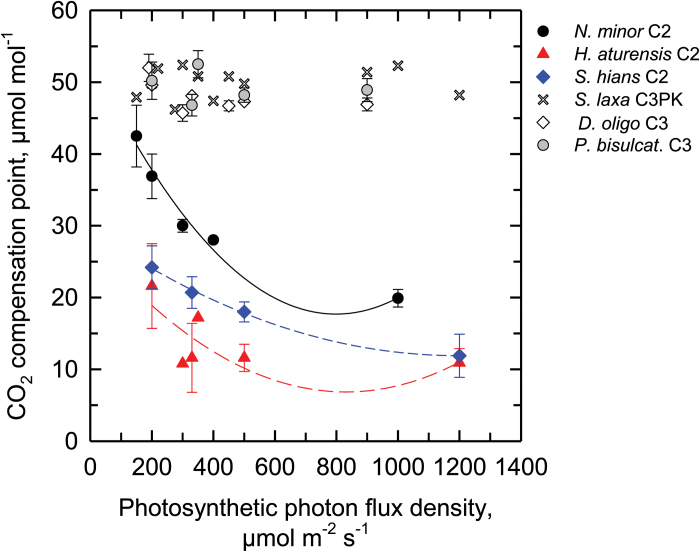
The response of the CO_2_ compensation point of *A* (Γ) as a function of measurement light intensity in the C_2_ species *Homolepis aturensis*, *Neurachne minor*, *Steinchisma hians*, the proto-Kranz species *Steinchisma laxum*, and the C_3_ species *Dicanthelium oligosanthes* and *Panicum bisulcatum*. Values of Γ were determined from the *A/C*_i_ curves used in the sequence of measurements to determine C_*_. Symbols are means ±SE (*n*=2–6), except where no error bar is shown, in which case *n*=1.

There is little observed shift to lower *C*_i_ in the high light response of *A* versus *C*_i_ in *H. aturensis*, such that all *A* versus *C*_i_ curves converge near a common intersection point (Fig. 5). In *S. hians* and *N*. *minor* there is a slight reduction by a *C*_i_ of ~5-7 µmol mol^−1^ in the high light response. There is no change in Γ in the C_3_ species with variation in light intensity (Fig. 7), while in each of the C_2_ species, Γ increases at the lower light intensity (Fig. 7). *Neurachne minor* exhibits the greatest increase in Γ as light declines, while the light response of Γ is negligible in *H. aturensis* above 300 µmol m^−2^ s^−1^. Notably, Γ of *N. minor* is twice that of *H. aturensis* across the range of light intensities.

### GDC BS specificity in C_2_ and C_4_ grasses probably results from changes in expression of a single GLDP gene

**Fig. 8.**
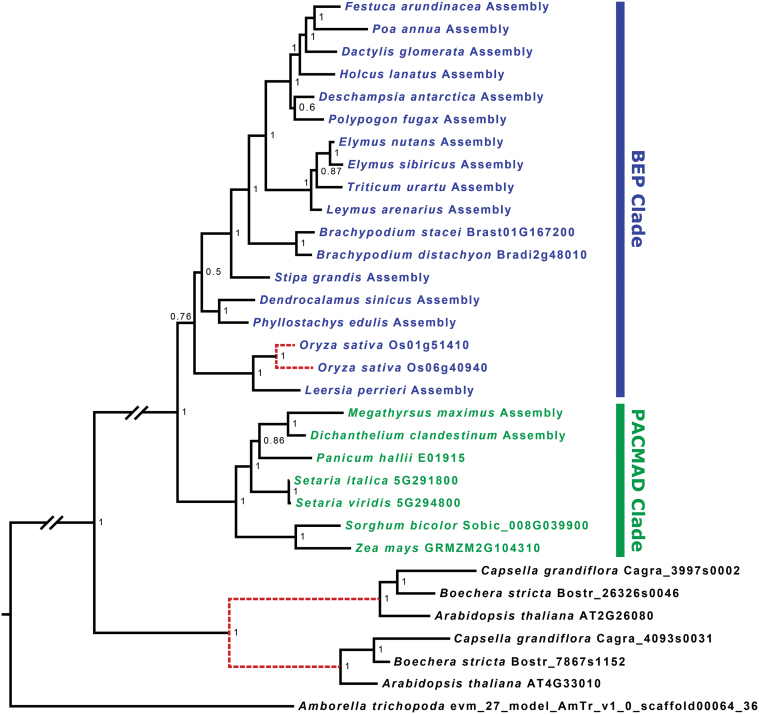
A Bayesian phylogenetic tree of GLDP nucleotide sequences from 24 grasses, three Brassicaceae species, and *Amborella*. Long branches between distantly related groups are condensed for visibility, denoted with a gap. Dashed red lines denote inferred gene duplication events. Numbers at nodes indicate posterior probability.

Phylogenetic analyses reveal that within the Poaceae, *O. sativa* is the only species examined with two gene copies encoding GLDP, and these sequences are most closely related to each other, even with inclusion of 14 additional BEP clade species (Fig. 8). We find no evidence of broader GLDP gene duplication or loss of gene copies in C_4_ grass species. Two paralogs encoding GLDH are present in all angiosperms, owing to an ancient duplication event (Supplementary Figs S7, S8). The two paralogs were treated independently as GLDH1 and GLDH2. Preliminary analysis indicates that there is a Poaceae-wide duplication only of GLDL, and targeted assemblies were made independently for each paralog, which we label GLDL1 and GLDL2 (Supplementary Fig. S9). During each assembly, mapped reads were scrutinized manually for evidence of additional paralogs, and none was detected. No reads were found for GLDL2 in the BEP grass *Dendrocalamus sinicus*, suggesting that it may have been lost in this species. Most other grass species possess both GLDL gene copies, with the exception of *Z. mays*, which also lacks GLDL2; *S. bicolor*, which shares a common C_4_ origin with *Z. mays*, has both paralogs. For all grass species, full GLDL1 and GLDL2 sequences from genomes and all assemblies which were completed to the start codon were strongly predicted to be mitochondrial-localized by TargetP (Emanuelsson *et al*., 2000; data not shown). This is consistent with targeting of the two copies in *Arabidopsis* (AT3G17240, AT1G48030; Fig. S9), which are both mitochondrial (Lutziger and Oliver, 2001; Rajinikanth *et al.*, 2007). A plastidial dihydrolipoyl dehydrogenase exists in land plants as well, but is more distantly related and likely dates to a much earlier duplication (Lutziger and Oliver, 2000; Rajinikanth *et al.*, 2007). Finally, GLDT is present as a single-copy gene in all species examined (Supplementary Fig. S10). While we find evidence of local duplication (GLDP, GLDH2) and conserved paralogs (GLDL), we find no evidence of C_4_ lineage loss of any GDC subunit genes.

## Discussion

Photorespiratory glycine shuttling exhibited by C_2_ species is considered to be the evolutionary bridge from C_3_ photosynthesis to C_4_ photosynthesis, based largely on studies from eudicot species, particularly *Flaveria* (Bauwe, 2011; Sage *et al.*, 2013; Schulze *et al.*, 2013; Mallmann *et al.*, 2014). It has been posited that C_2_ photosynthesis may serve a bridging role to C_4_ photosynthesis in grasses as well (Schulze *et al.*, 2013), although the timing of trait acquisition, to include GDC BS cell specificity and abundance, has been proposed to differ between eudicots and monocots (Williams *et al.*, 2013). Here, we evaluated photosynthetic pathway characteristics and cellular features of BS and M cells in *H. aturensis*, a candidate C_2_ species which branches in a position sister to a C_4_ clade in the subtribe Arthropogoninae. We used comparative studies with confirmed C_2_ grasses, *S*. *hians* and *N*. *minor*, and the C_3_ grass *S*. *laxum* to facilitate our classification of the photosynthetic type of *H*. *aturensis*. In addition, we conducted a phylogenetic study of genes for the GDC subunits to test the Schulze *et al.* (2013) inference that the evolution of BS-specific GDC expression in C_4_ grasses was similar to that in *Flaveria*. From our physiological and structural results, we conclude that *H*. *aturensis* is indeed a C_2_ species, supporting a hypothesis that the photorespiratory glycine shuttle is a bridge to C_4_ photosynthesis in grasses in the subtribe Arthropogoninae. The characterization of *S*. *laxum* and *S*. *hians* also allows us to conclude that activation of the BS cells during transition from C_3_ to C_2_ in grasses in the subtribe sister to the Arthropogoninae is similar to what has been reported for eudicots with respect to BS organelle positioning and organelle and GDC enrichment (Muhaidat *et al.*, 2011; Sage *et al.*, 2013). Finally, our phylogenetic and immunohistochemical data are consistent with the notion that BS GDC in C_2_ and C_4_ grasses results from changes in expression levels of a single GLDP gene in M and BS cells.

*Homolepis aturensis* exhibits characteristic features common to species that concentrate photorespired CO_2_ in BS cells using the C_2_ metabolic cycle. One of the key proteins essential for GDC activity, GLDP, localizes almost exclusively in BS mitochondria in *H*. *aturensis* and *S*. *hians*. This pattern is ubiquitous in C_2_ eudicots (Hylton *et al.*, 1988; Voznesenskaya *et al.*, 2001; Ueno and Sentoku, 2006; Voznesenskaya *et al.*, 2010; Muhaidat *et al.*, 2011; T.L. Sage *et al.*, 2011). A second characteristic of C_2_ species is an abundance of Rubisco in M and BS cells (Monson *et al.*, 1984). Rubisco is abundant in M and BS cells in *H*. *aturensis* and *S*. *hians*, in contrast to the typical C_3_ pattern (high M Rubisco; low BS Rubisco) and C_4_ pattern (Rubisco only in BS cells). Lastly, the number of M cells between BS cells in *H*. *aturensis* and *S*. *hians* results in a reduced M:BS ratio and increased vein density common to C_2_ species (reviewed in Sage *et al.*, 2012). These features promote rapid flux of photorespiratory metabolites between M and BS compartments (Monson and Rawsthorne, 2000), improve water relations under high photorespiratory conditions (Osborne and Sack, 2012), and facilitate an increase in the volume of leaf tissue where photorespired CO_2_ is concentrated around Rubisco in C_2_ species.

As the refixed fraction of photorespiratory CO_2_ increases, C_*_ (and Γ) declines (von Caemmerer, 2000). The effectiveness of the C_2_ process in *H*. *aturensis* as well as *S*. *hians* is reflected in the Γ and C_*_ values that are at the lower range of values reported for C_2_ species (Holaday *et al.*, 1984; Monson *et al.*, 1984; Ueno *et al.*, 2003; Vogan *et al.*, 2007; Voznesenskaya *et al.*, 2010; T.L. Sage *et al.*, 2011, 2013). Values of Γ and C_*_ below 15ppm indicate either that a C_4_ cycle is active to complement the C_2_ cycle, or that the CO_2_ trap in the inner BS is particularly effective at recapturing the photorespired CO_2_ (von Caemmerer, 2000). *Steinchisma hians* has weak to negligible C_4_ cycle activity (Edwards *et al.*, 1982), and cellular characteristics of organelle orientation in BS cells may explain the low C_*_ in *H*. *aturensis* and *S*. *hians*. The organelle-enriched BS cells of these two species exhibit polarity in organelle positioning such that almost all peroxisomes and GDC-containing mitochondria, and over half of the chloroplasts are situated adjacent to the vascular tissue. This arrangement of BS organelles is posited to be particularly effective in enhancing recapture efficiency of photorespired CO_2_ before it can escape the BS cell in C_2_ species (Rawsthorne, 1992). One notable feature we observed is that the parallel orientation of the centripetal BS chloroplasts to the inner wall in *S*. *laxum* shifts to a perpendicular orientation in *S*. *hians*. A similar perpendicular chloroplast orientation is present in *H*. *aturensis*. This pattern allows for packaging of the more numerous, larger chloroplasts in the centripetal position, which could be important for increasing the surface area for refixing photorespired CO_2_ from adjacent mitochondria. Moreover, BS mitochondria are physically surrounded by chloroplasts in *S*. *hians* and *H*. *aturensis*, and these close physical associations have been proposed to enhance refixation of photorespired CO_2_ (Brown *et al.*, 1983).

The fine structure of C_2_ BS cells is defined as C_2_ Kranz, reflecting a view that this photosynthetic carbon-concentrating mechanism is associated with its own enabling Kranz-like structure (Sage *et al.*, 2014). Multiple convergence of C_2_ Kranz in eudicots and grasses is strong evidence that this particular BS anatomy is specifically adapted for the C_2_ pathway. The earliest recognizable subcellular events that ‘increase the accessibility’ (Grass Phylogeny Working Group II, 2012) of C_2_ Kranz from C_3_ in eudicots are an enhancement in numbers and size of mitochondria per BS cell, and positioning of these organelles from the centrifugal C_3_ position to the centripetal BS pole. The BS chloroplast numbers also increase in tandem with alterations in mitochondria placement, along with a rearrangement of many, but not all, BS chloroplasts from the centrifugal to centripetal pole (Muhaidat *et al.*, 2011; Sage *et al.*, 2013; Voznesenskaya *et al.*, 2013). The anatomy associated with these earliest subcellular events has been termed proto-Kranz (Muhaidat *et al.*, 2011; Sage *et al.*, 2013). The transition to full C_2_ BS patterns from proto-Kranz in eudicots results from further amplification in centripetal mitochondria volume (size and numbers) and relocation of a greater fraction of enlarged chloroplasts to the centripetal pole (Muhaidat *et al.*, 2011; Sage *et al.*, 2013). Proto-Kranz and the shift to C_2_ Kranz occurs with increasing vein density in *Flaveria* and *Heliotropium* (Muhaidat *et al.*, 2011; Sage *et al.*, 2013).

The subcellular framework of C_3_ *S*. *laxum* BS cells and subsequent changes to that configuration from *S*. *laxum* to C_2_ *S*. *hians* are similar to those observed in eudicots, supporting a hypothesis that proto-Kranz facilitates the C_3_ to C_2_ transition (Sage *et al.*, 2014). In comparison with the C_3_ species *D*. *oligosanthes* and *P*. *bisulcatum*, mitochondria and peroxisomes are situated along the centripetal poles of BS cells in *S*. *laxum*, classifying this species as proto-Kranz. Previous characterizations of proto-Kranz species have not presented data on peroxisomes, and this additional focus in the present study provides critical information on the positioning of the other organelle involved in C_2_ photosynthesis in the BS. A 3-fold increase in mitochondrial volume and corresponding increase in GDC, as well as a 2.5-fold increase in peroxisome volume, and 2-fold increase in chloroplast volume accompany the transition to the C_2_ BS pattern in *S*. *hians*. Also, as observed in eudicots, the increase in BS chloroplast volume in *S*. *hians* from proto-Kranz is associated with more of these organelles in the centripetal location. Notably, although the number and size of mitochondria per BS cell area are lower in *S*. *laxum* than in *S*. *hians*, the GLDP label intensity is similar per mitochondrion and significantly higher than that observed in the C_3_ grasses. These results indicate that increased BS GDC per mitochondrion is also a functionally important development early in C_2_ evolution in grasses. The enhanced BS GDC density per mitochondrion in *S*. *laxum* is present in tandem with C_3_-like values of vein density. The C_2_ levels of BS GDC in *S*. *hians* are present in high vein density leaves. The high levels of BS GDC in *S*. *laxum* and *S hians* contrasts with the theoretical predictions of Williams *et al.* (2013) who modeled C_4_ evolution in eudicots and monocots based on observed patterns of trait acquisition in C_3_–C_4_ intermediates. For monocots, they predicted that an increase in vein density preceded enhanced GDC specificity and abundance in BS cells. However, their model relied on a relatively small data set with significant gaps. The results here indicate that C_2_ evolution in grasses follows a pattern more typical of eudicots, which the model of Williams *et al.* (2013) may support when reparameterized with a richer data set.

In *Neurachne*, as in many other grasses, the mestome sheath cell is the site of the Calvin–Benson cycle in C_4_ species (Hattersley and Browning, 1981; Hattersley *et al.*, 1986; Dengler and Nelson, 1999; Edwards and Voznesenskaya, 2011). We demonstrate that the mestome sheath cells in the C_2_ species *N*. *minor* are functionally similar to the C_2_ BS cells of *H*. *aturensis* and *S*. *hians* because GDC is almost exclusively located in organelle-enriched mestome sheath cells in the high vein density leaves. However, unlike *H*. *aturensis* and *S*. *hians*, there is no polarized orientation of organelles in the GDC-enriched mestome sheath cells of *N*. *minor* (Hattersley *et al.*, 1986; this study), indicating that *N. minor* utilizes a different strategy from *H*. *aturensis* and *S*. *hians* to trap photorespired CO_2_. In *Neurachne*, as in many other grasses, the thick mestome sheath cell wall with a suberized lamella becomes the trap (Hattersley and Browning, 1981; Hattersley *et al.*, 1986; Dengler and Nelson, 1999; Edwards and Voznesenskaya, 2011). The C_2_ species *Alloteropsis semialata* ssp *semialata* has a similar C_2_ Kranz anatomy to *N*. *minor*; GDC levels are highest in mestome sheath cells with a suberized lamella and the abundant organelles are equally partitioned within those cells (Hattersley and Browning, 1981; Ueno and Sentoku, 2006). Intriguingly, although organelle orientation in mestome sheath cells is not important in the evolution of C_2_ photosynthesis in *N*. *minor*, chloroplasts do have a centrifugal orientation in C_4_ *Neurachne* species (Hattersley *et al.*, 1986). Qualitative observations on C_3_ *Neurachne* species indicate that some of the C_3_ species have enhanced numbers of chloroplasts and mitochondria in mestome sheath cells (Hattersley *et al.*, 1986), leading us to posit that organelle and GDC enrichment may have been important during the early stages of C_2_ evolution in the genus.

The evolutionary transition from C_3_ to C_2_ has been proposed first to involve a change in cell type-specific expression of GDC from M to BS in tandem with a loss of M GDC (Bauwe, 2011). A comparison of the cellular site of GDC expression in proto-Kranz *S*. *laxum* with that of the sister species *S*. *hians* indicates that the severe reduction in M GDC in the C_2_ species is preceded by increased expression in BS cells. This is consistent with patterns observed in the eudicots *Flaveria* and *Heliotropium*, and supports a model of gradual GDC loss in M cells following a physiological activation of the BS (Muhaidat *et al.*, 2011; Sage *et al.*, 2013; Schulze *et al.*, 2013). C_3_ species of *Flaveria* contain two copies of the gene encoding GLDP resulting from gene duplication (Schulze *et al.*, 2013). One of these is BS dominant in expression and the second is expressed ubiquitously throughout the leaf in C_3_ *Flaveria* (Schulze *et al.*, 2013). During the evolution of C_4_ photosynthesis, the loss of M GDC function in *Flaveria* resulted from pseudogenization of the gene coding for the ubiquitously expressed GLDP (Schulze *et al.*, 2013). Schulze *et al.* (2013) speculated that BS-dominant GLDP expression arose in a similar manner in C_4_ grasses, because *O. sativa* (C_3_ BEP clade) has two GLDP genes, but *Z. mays* and other C_4_ species in the C_4_ PACMAD clade have only one. To provide an understanding of the evolution of BS-specific GDC expression in C_2_ and C_4_ grasses, we conducted phylogenetic analyses of genes encoding GLDP using 17 BEP and seven PACMAD grass species, three Brassicaceae species, and *Amborella* as outgroup. Our analyses demonstrate that, with the exception of *O. sativa*, all grasses have one copy of GLDP. The two copies of the genes encoding GLDP in rice are more closely related to each other than to any other GLDP gene included in the analysis and therefore represent a local duplication. Combined, the phylogenetic and immunohistochemical observations on C_3_ and C_4_ PACMAD species are consistent with a hypothesis that BS-dominant quantities of GDC in C_2_ and C_4_ grasses resulted from modifications in regulatory mechanisms controlling the levels of expression of a single GLDP gene present in M and BS cells; C_3_ species have high levels of M GDC and low levels of BS GDC, and the opposite pattern is present in the C_2_ and C_4_ species. Mesophyll tissue specificity of phosphoenolpyruvate carboxylase has evolved through modification of *cis*-regulatory elements in C_4_ *Flaveria* (Gowik *et al.*, 2004). Studies examining promoter regions of the GLDP subunits in closely related C_3_, C_2_, and C_4_ species should provide insights into the evolution of the regulatory mechanisms that confer the requisite expression patterns for C_2_ and subsequently C_4_ photosynthesis in grasses.

Since it is conceivable that BS specificity of the GDC complex arose via duplication and pseudogenization of one of the other subunits, we also included analyses for GLDL, GLDH1 and GLDH2, and GLDT. In land plants, GLDH1 functions in photorespiration and GLDH2 is associated with C1 metabolism (Rajinikanth *et al.*, 2007). We found no evidence of broad duplication for either of the ancient GLDH copies or GLDT in the grasses; however, GLDL is encoded by two conserved paralogs in most grass species, and may have arisen from the whole-genome duplication in the ancestor of the grass family. GLDL is the only GDC subunit gene for which we find evidence of subfunctionalized and conserved paralogs analogous to the two GLDP copies in *Flaveria*. There is, however, no evidence that either of these copies has been lost or pseudogenized (e.g. via nonsense or frameshift mutation) in any C_4_ species except *Z. mays*, which has a local duplication of GLDL1 and lacks a gene encoding GLDL2. Yet it is unlikely that this loss was involved in the evolution of C_4_ photosynthesis, as GLDL2 is present in *S. bicolor*, which shares a common C_4_ origin with maize (Grass Phylogeny Working Group II, 2012). The two GLDL paralogs in grasses may be partially reduntant with one another (Rajinikanth *et al.*, 2007). The nature of the subfunctionalization of GLDL which resulted in the evolutionary retention of two paralogs in Poaceae is not known, but our results indicate that these paralogs did not play a role in the evolution of C_4_ photosynthesis analogous to the GLDP paralogs in *Flaveria*. It is similarly unlikely that the two ancestral copies of GLDH played a role in C_4_ evolution analogous to that of GLDP in *Flaveria* as both are present in all grass species examined here, and only GLDH1 plays a role in photorespiration (Rajinikanth *et al.*, 2007).

## Conclusion

Evolution of C_2_ photosynthesis in the grasses and eudicots is conferred by organelle enrichment, BS- or mestome sheath-dominant GDC accumulation, and centripetal positioning of organelles when the BS is the carbon-concentrating tissue. In *Steinchisma*, the earliest recognized subcellular event that facilitates C_2_ photosynthesis is the placement of BS mitochondria and peroxisomes exclusively to the centripetal pole. This feature, also present in eudicots, is posited to set in motion a feed-forward facilitation cascade that leads to C_2_ and subsequently C_4_ photosynthesis (Sage *et al.*, 2013). How and why changes in BS or mestome sheath GDC levels and organelle volume, and BS organelle positioning were initiated during the early stages of C_2_ evolution in grasses remains a mystery. Identification of mechanisms controlling these processes should be a primary focus of research. The Arthropogoninae, the subtribe containing *Steinchisma* (Otachyrinae), and Neurachninae will be key to these future studies.

## Supplementary data

Supplementary data are available at *JXB* online.

Table S1. List of species studied and source of species.

Table S2. Species for which RNA-seq data were downloaded and assembled for phylogenetic analysis of GDC subunits.

Table S3. Anatomical parameters of C_3_, C_2_, and C_4_ leaves.

Table S4. Organelle distribution in bundle sheath cells of C_3_ and C_2_ species.

Table S5. Quantification of organelle numbers, size, and density of gold labeling (GLDP) in mesophyll and bundle sheath cells of C_3_, C_2_, and C_4_ leaves.

Figure S1. Light micrographs of C_3_ and C_4_ species.

Figure S2. Bundle sheath cell ultrastructure of C_3_ and C_4_ species.

Figure S3. Leaf structure and anatomy and immunolocalization of GLDP in *N. minor*.

Figure S4. Immunolocalization of GLDP in M and BS cells of C_3_ and C_4_ species.

Figure S5. Immunolocalization of Rubisco large subunit in M and BS cells of C_3_ and C_2_ species.

Figure S6. Immunolocalization of Rubisco large subunit in M and BS cells of C_3_ and C_4_ species.

Figure S7. A Bayesian phylogenetic tree of GLDH1.

Figure S8. A Bayesian phylogenetic tree of GLDH2.

Figure S9. A Bayesian phylogenetic tree of GLDL.

Figure S10. A Bayesian phylogenetic tree of GLDT.

Supplementary Data
