# Supplementary material for: C3–C4 intermediacy in grasses: organelle enrichment and distribution, glycine decarboxylase expression, and the rise of C2 photosynthesis
Source: J Exp Bot. 2016 Apr 12;67(10):3065–78. doi: 10.1093/jxb/erw150 (PMC4867898; doi:10.1093/jxb/erw150)
Supplement: Supplementary Data [file supp_67_10_3065__index.html]

C3–C4 intermediacy in grasses: organelle enrichment and distribution, glycine decarboxylase expression, and the rise of C2 photosynthesis — C3–C4 intermediacy in grasses: organelle enrichment and distribution, glycine decarboxylase expression, and the rise of C2 photosynthesis — Supplementary Data 

# C3–C4 intermediacy in grasses: organelle enrichment and distribution, glycine decarboxylase expression, and the rise of C2 photosynthesis

## Supplementary Data

Data files

- supplementary\_tables\_S1\_S5.pdf - Supplementary Data
- supplementary\_figures\_S1\_S10.pdf - Supplementary Data
