# Supplementary material for: C3–C4 intermediacy in grasses: organelle enrichment and distribution, glycine decarboxylase expression, and the rise of C2 photosynthesis
Source: J Exp Bot. 2016 Apr 12;67(10):3065–78. doi: 10.1093/jxb/erw150 (PMC4867898; doi:10.1093/jxb/erw150)
Supplement: Supplementary Data [file supp_erw150_supplementary_tables_S1_S5.pdf]

Supplementary Table S1. List of species studied in this paper and source of species.

Species	Seed/plant source
Dicanthelium oligosanthes	Elizabeth Kellogg, Donald Danforth Plant Science Center St. Louis Missouri, USA
Panicum bisulcatum	Mitsutaka Taniguchi, Graduate School of Bioagricultural Sciences, Nagoya University, Nagoya, Japan
Steinchisma laxum	Elizabeth Kellogg, Donald Danforth Plant Science Center St. Louis Missouri, USA
Steinchisma hians	Elizabeth Kellogg, Donald Danforth Plant Science Center St. Louis Missouri, USA
Homolepis aturensis	Costa Rica, 9°39’27.4”N 82°45’08.0”W
Setaria viridis	Thomas Brutnell, Donald Danforth Plant Science Center St. Louis Missouri, USA
Panicum virgatum	Summerhill Nursery, Toronto, Ontario, Canada

Supplementary Table S2. Additional species for which RNA-seq data was downloaded and assembled for phylogenetic analysis of the GDC subunit genes, with NCBI SRA run IDs.

SPECIES	NCBI SRA RUN ID	SEQUENCING DEPTH
Dactylis glomerata	SRR1632251	325.7 Million Paired-End Reads
Dendrocalamus sinicus	SRR2543019	27.4 Million Single-End Reads
Deschampsia antarctica	SRR1162371	29.7 Million Paired-End Reads
Dichanthelium clandestinum	SRR1158317-8	112.2 Million Single-End Reads
Elymus nutans	SRR1336283	77.1 Million Paired-End Reads
Elymus sibiricus	SRR1344836	76.7 Million Paired-End Reads
Festuc aarundinacea	SRR2099408	55.1 Million Paired-End Reads
Holcus lanatus	ERR294017	111.7 Million Paired-End Reads
Leersia perrieri	SRR1171869	403.9 Million Paired-End Reads
Leymus arenarius	SRR1812885	141.3 Million Paired-End Reads
Megathyrsus maximus	SRR821833	199.14 Million Paired-End Reads
Phyllostachys edulis	SRR2035327	144.3 Million Paired-End Reads
Poa annua	SRR1633980	258.7 Million Paired-End Reads
Polypogon fugax	SRR1721962	27.2 Million Single-End Reads
Stipa grandis	SRR1740003	66.0 Million Paired-End Reads
Triticum urartu	ERR420228-9	203.3 Million Paired-End Reads

Supplementary Table S3. Anatomical characteristics of C_3, C_2, and C_4 leaves. Values represent mean ± SE, n=3 plants per species. Values followed by the same letter are not different from one another as determined by one-way ANOVA and the Tukey's post-hoc test.

	Species	Mesophyll (%)	Bundle Sheath (%)	Mesophyll:Bundle Sheath	Distance between adjacent minor veins (µm)	mesophyll cells # between minor veins
C_3 species	*Dicanthelium oligosanthes*	40.0±2 a, b	7.2±0.5 a	5.7±0.5 a	206±6 a	6.6±0.4 a
	Panicum bisulcatum	45 ±1 a	10.0±1.2 a	5.4±1.0 a	240±9 a	7.4±2.9 a
C_3-proto-Kranz	*Steinchisma laxum*	39±2 a, b	18.0±1.5 b	2.4±0.4 b	188 ±10 a, c	6.4±0.3 a
C_2 species	*Homolepis aturensis*	42±2 a	18.7±1.1 b	2.1±0.2 b	139±7 a, c	3.9±0.4 a, c
	Steinchisma hians	40±1a, b	21.3±1.3 b	2.2±0.1 b	129±2 a, c	4.3±0.2 a, c
	Neurachne minor	47±6 b	8.5±1.3 a	6.5±1.5 a	100±19	2.5±0.3
C_4 species	*Panicum virgatum*	35±1 b	20.9±1.0 b	1.8±0.1 b	123±3 b	2.0±0.0* b, c
	Setaria viridis	46±1 a	10.1±0.7a	4.7±0.4 a	84±4 b, c	2.0±0.2 b, c

* There is one colorless cell between the two mesophyll cells of *P. virgatum.*

Supplementary Table S4. Characteristics of bundle sheath organelle positioning: Values represent mean ± SE, n=3 plants per species. Values followed by the same letter are not different from one another as determined by one-way ANOVA and the Tukey's post-hoc test.

	Species	Coverage of Inner Half of Cell Area, %	Proportion in Inner Half of Cell Area, %
	Chloroplasts		
C_3 species	*Dicanthelium oligosanthes*	1.3±0.2 a	38.6±6.0 a
	Panicum bisulcatum	3.4±0.3 b, c	47.5±3.2 a
C_3-Protokranz	*Steinchisma laxum*	2.1±0.2 a, c	52.5±2.7 a, b
C_2 species	*Steinchisma hians*	7.2±0.5 b	60. 6±2.8 b
	Homolepis aturensis	5.1±0.5 b	62.2±2.6 b
	Mitochondria		
C_3 species	*Dicanthelium oligosanthes*	0.20±0.03 a	68.8±4.7 a
	Panicum bisulcatum	0.18±0.03 a	65.2±5.8 a
C_3-Protokranz	*Steinchisma laxum*	0.27±0.02 a	98.3±0.9 b
C_2 species	*Steinchisma hians*	0.81±0.07 b	99.6±0.2 b
	Homolepis aturensis	0.73±0.06 b	98.8±0.6 b
	Peroxisomes		
C_3 species	*Dicanthelium oligosanthes*	0.03±0.01	18.9±7.1 a
	Panicum bisulcatum	0.10±0.02 a	41.4±7.6 a
C_3-Protokranz	*Steinchisma laxum*	0.10±0.01 a	80.0±7.4 b
C_2 species	*Steinchisma hians*	0.27±0.04 b	92.9±4.6 b
	Homolepis aturensis	0.22±0.03 b	91.4±4.0 b

Supplementary Table S5. Organelle parameters in the mesophyll and bundle sheath cells of nine species of grasses in the Panicoideae. Values represent means ± SE. Values followed by the same letter are not different from one another as determined by one-way ANOVA and the Tukey's post-hoc test.

		Mitochondria Planar Area/Planar Cell Area, %	Mitochondria number /Planar Cell Area, $\mu m^{-2} \times 10^{-3}$	Density of Gold Labeling/ Planar Mitochondria Area, μm^{-2}	Density of Gold Labeling/ Planar Call Area, μm^{-2}	Peroxisome Planar Area/Planar Cell Area, %	Peroxisome number /Planar Cell Area, $\mu m^{-2} \times 10^{-3}$	Chloroplast Planar Area/Planar Cell Area, %	Chloroplast number /Planar Cell Area, $\mu m^{-2} \times 10^{-3}$
	**Mesophyll Cells**								
C_3 species	*Dicanthelium oligosanthes*	0.50±0.05 a	30±3 ac	63.1±2.5 a	0.314±0.03 a	0.323±0.038 a	13±1 ab	26.9±1.8 a	26±1 a
	Panicum bisulcatum	0.29±0.04 a	16±2 b	76.4±4.8 a	0.194±0.02 a	0.333±0.050 a	11±2 ab	28.7±1.7 a	27±2 a
C_3-Protokranz	*Steinchisma laxum*	0.39±0.03 ab	34±3 a	83.5±5.5 a	0.304±0.02 a	0.588±0.056 a	21±2 a	30.3±1.6 a	33±2 ab
C_2 species	*Homolepis aturensis*	0. 30±0.06 bc	20±bc	1.3±0.3 b	0.005±0.00 b	0.376±0.059 a	7±1 b	17.7±1.2 b	27±1 a
	Steinchisma hians	0.39±0.04 ab	37±3 a	1.5±0.4 b	0.007±0.002b	0.414±0.052 a	20±3 a	28.2±1.6 a	48±2 b
	Neurachne minor	0.59±0.07	22±3	2.2±0.3	0.012±0.002	0.011±0.002	3±1	19.6±1.6	28±2
C_4 species	*Setaria viridis*	0.13±0.02c, d	12±3 b	0.7±0.3 b	0.001±0.000b	0.005±0.003 b	1±0.00 c	14.6±1.1 b	11±1
	Panicum virgatum	0.09±0.02 d	12±2 b	4.61±1.1 b	0.007±0.002b	0.002±0.002 b	3±0.00 c	12.3±0.9 b	22±2 a
	**Bundle Sheath Cells**								
C_3 species	*Dicanthelium oligosanthes*	0.28±0.03 a	30±3 ac	6.0±0.8 a	0.02±0.00 a	0.044±0.016 a	3±1 a	3.8±0.4 a	12±1 a
	Panicum bisulcatum	0.24±0.03 a	25±4 a	33.7±3.2cd	0.08±0.01 ab	0.152±0.033 b, c	8±2 bcd	7.6±0.5 bc	22±1 b
C_3-Protokranz	*Steinchisma laxum*	0.27±0.02 a	20±2 a	109.9±6.2 b	0.27±0.02 bc	0.105±0.015 b, c	4±1 a, c	4.9±0.5 ab	11±1 a
C_2 species	*Homolepis aturensis*	0.73±0.06 b	32±3 ac	47.2±2.1 cd	0.35±0.03 c	0.227±0.027 b	7±1 c, d	8.3±0.7 bc	16±1 ac
	Steinchisma hians	0.81±0.06 b	53±3 b	92.2±3.7 b	0.74±0.05 d	0.266±0.040 b	12±1 d	11.2±0.8 ce	20±1 bc
	Neurachne minor	6.95±0.80	183±0.018	49.6±1.7	3.39±0.34	0.002±0.001	5±2	32.5±2.4	44±3
C_4 species	*Panicum virgatum*	0.74±0.07 b	40±4 bc	52.3±1.7 c	0.38±0.03 c, d	0.069±0.014 ac	3±1 ab	18.5±1.5 de	18±1 bc
	Setaria viridis	0.64±0.08 b	30±3 ac	28.0±2.4 ad	0.17±0.02 b	0.062±0.017 ac	4±1 a, bc	41.8±1.7 d	21±1 b
