# Supplementary material for: C3–C4 intermediacy in grasses: organelle enrichment and distribution, glycine decarboxylase expression, and the rise of C2 photosynthesis
Source: J Exp Bot. 2016 Apr 12;67(10):3065–78. doi: 10.1093/jxb/erw150 (PMC4867898; doi:10.1093/jxb/erw150)

Supplementary Figure S1. Leaf cross sections of (A) *Dicanthelium oligosanthes* (Diol, C_3); (B) *Panicum bisulcatum* (Pabi, C_3); (C) *Setaria viridis* (Sevi, C_4); (D) *P. virgatum* (Pavi, C_4). M, mesophyll; asterisk marks bundle sheath. Bars = 50 µm.

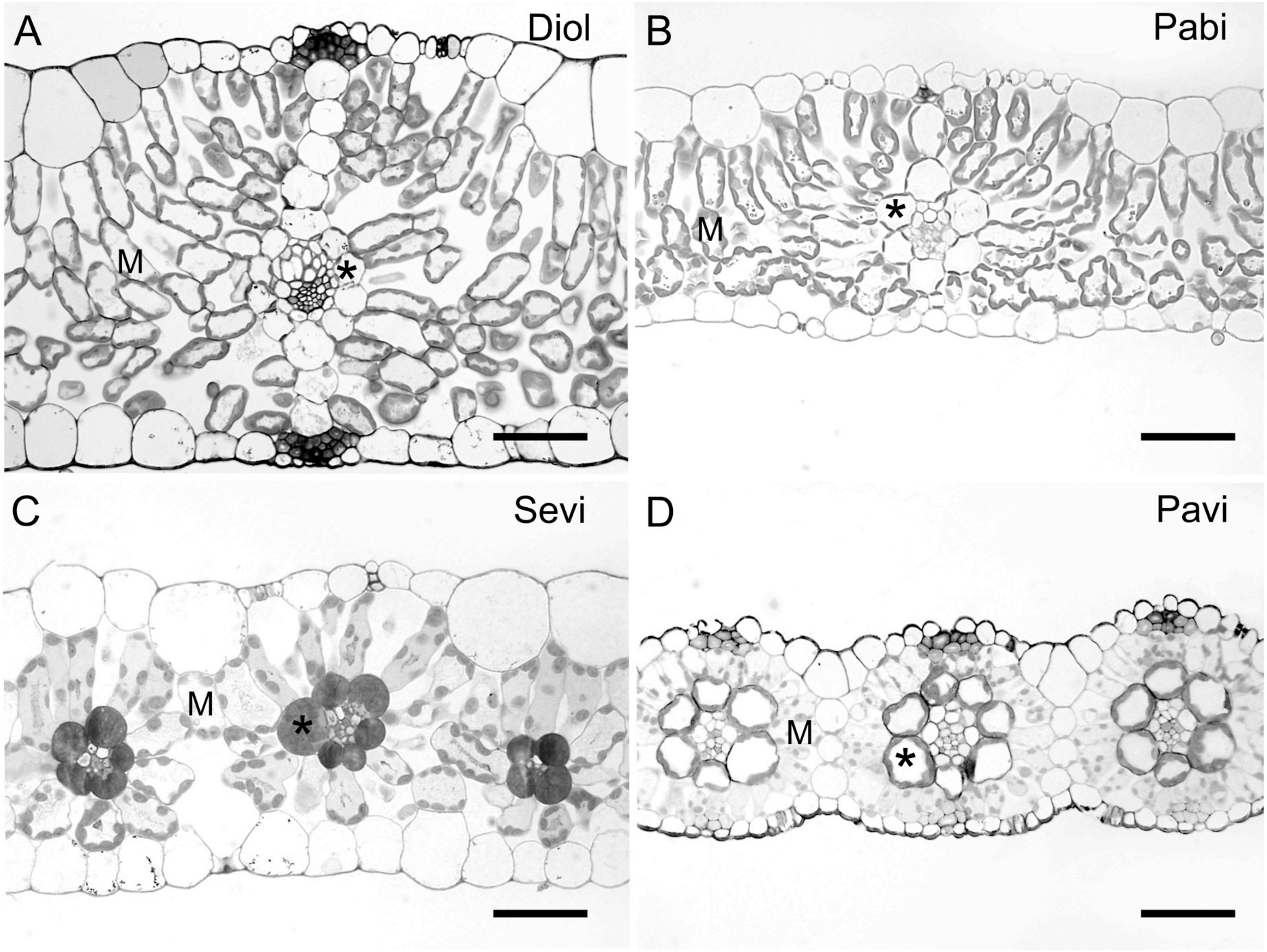

Supplementary Figure S2. Ultrastructure of C_3 and C_4 bundle sheath cells from (A) *Dicanthelium oligosanthes* (Diol, C_3); (B) *Panicum bisulcatum* (Pabi, C_3); (C) *P. virgatum* (Pavi, C_4) and (D) *Setaria viridis* (Sevi, C_4). BS, bundle sheath; M, mesophyll; VB, vascular tissue; c, chloroplast; arrowheads mark mitochondria. Bars = 2 µm.

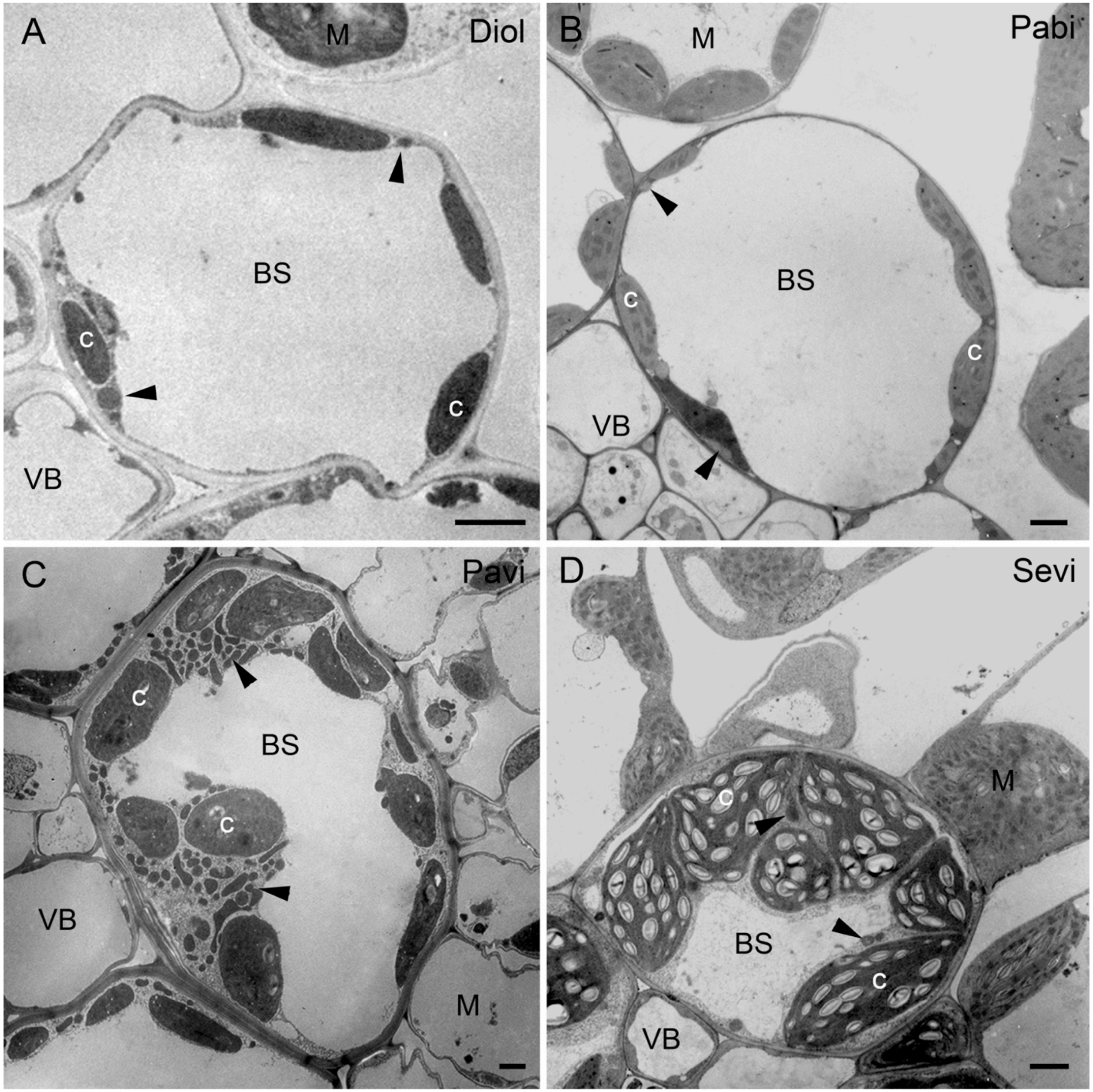

Supplementary Figure S3. Leaf structure and immunogold labeling of GLDP in *Neurachne minor*. (A-B) low and high magnification image of leaf illustrating mestome sheath cells (white asterisk). (C) Organelle enriched mestome sheath cell. (D-E) immunogold labeling for GLDP in mestome sheath (D) and mesophyll (E). M, mesophyll; BS, bundle sheath; VB, vascular tissue; c, chloroplast; arrowheads mark mitochondria; black asterisk marks bundle sheath. Bars = 50 μm for A and B; 2 μm for C; 500 nm for D and E.

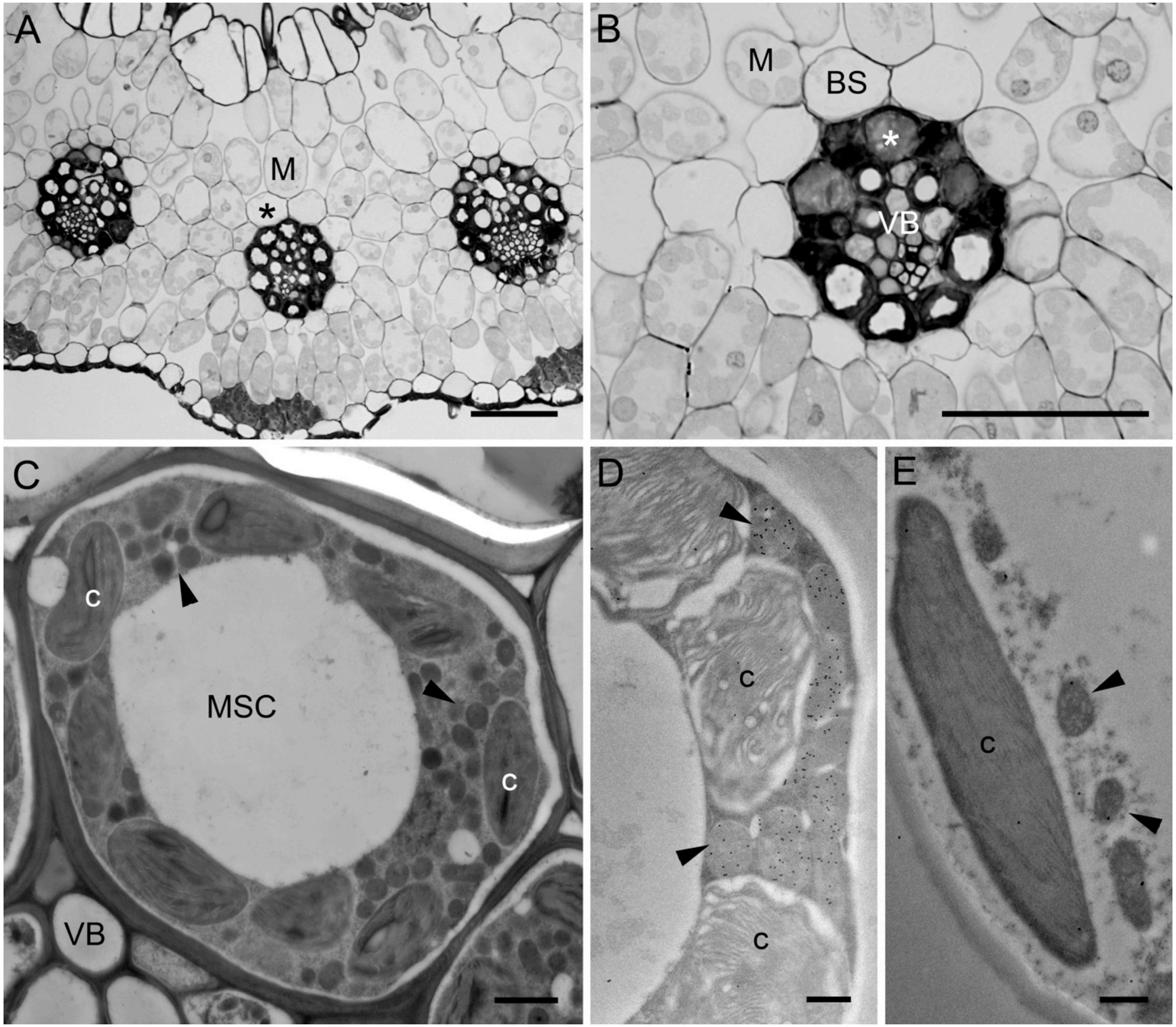

Supplementary Figure S4. Immunogold labeling of GLDP in the bundle sheath and mesophyll cells of (A-B) *Dicanthelium oligosanthes* (C_3); (C-D) *Panicum bisulcatum* (C_3), (E-F) *P. virgatum* (C_4) and (G-H) *Setaria viridis* (C_4) . p, peroxisomes; c, chloroplasts; asterisk marks mitochondria. Bars = 500 nm.

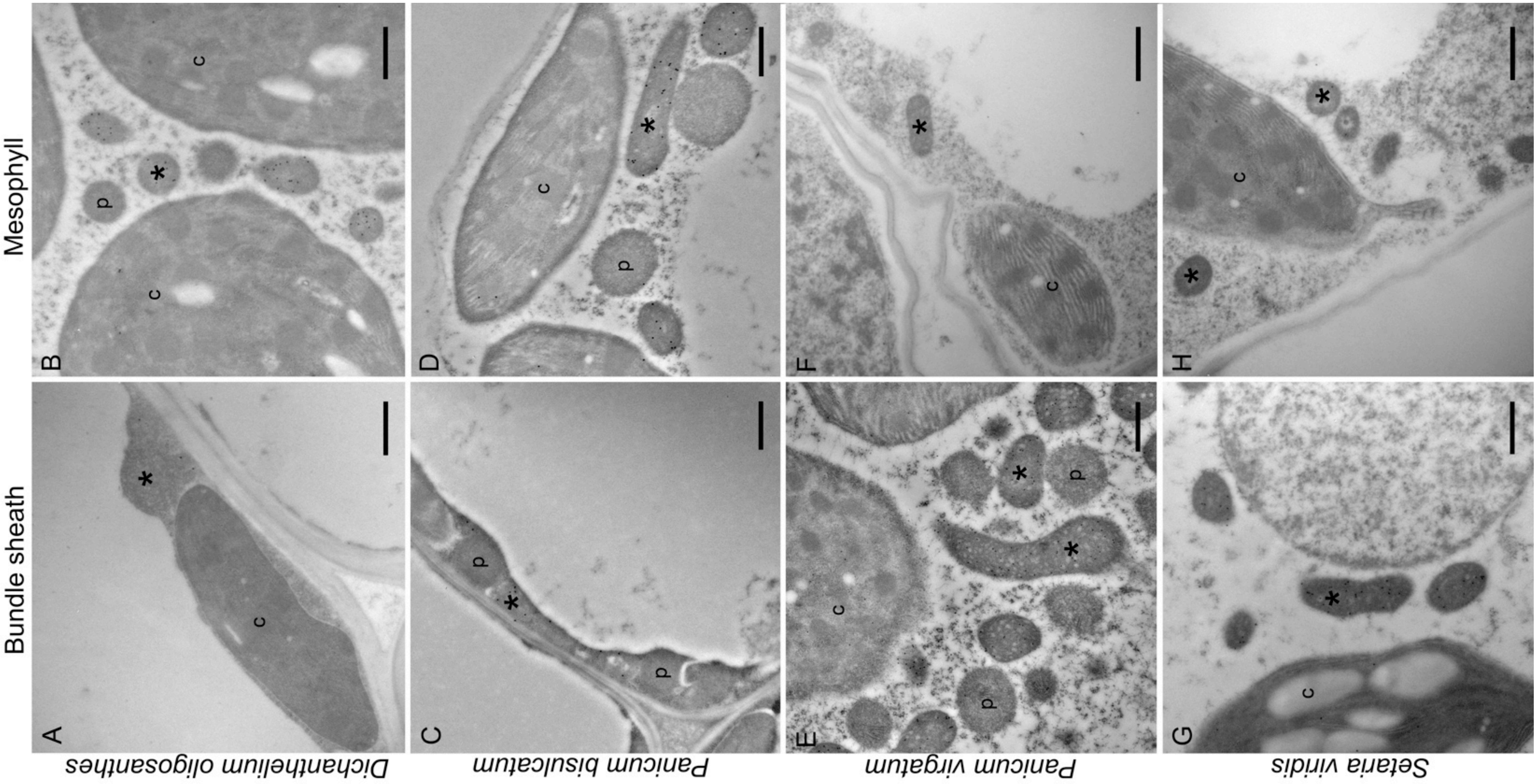

Supplementary Figure S5. Immunogold labeling for Rubisco large subunit in the bundle sheath and mesophyll cells of (A-B) *Homolepis aturensis*, (C-D) *Steinchisma laxum*, (E-F) *S. hians*, and (G-H) *Neurachne minor*. c, chloroplast; p, peroxisome; asterisk marks mitochondria. Bars = 500 nm.

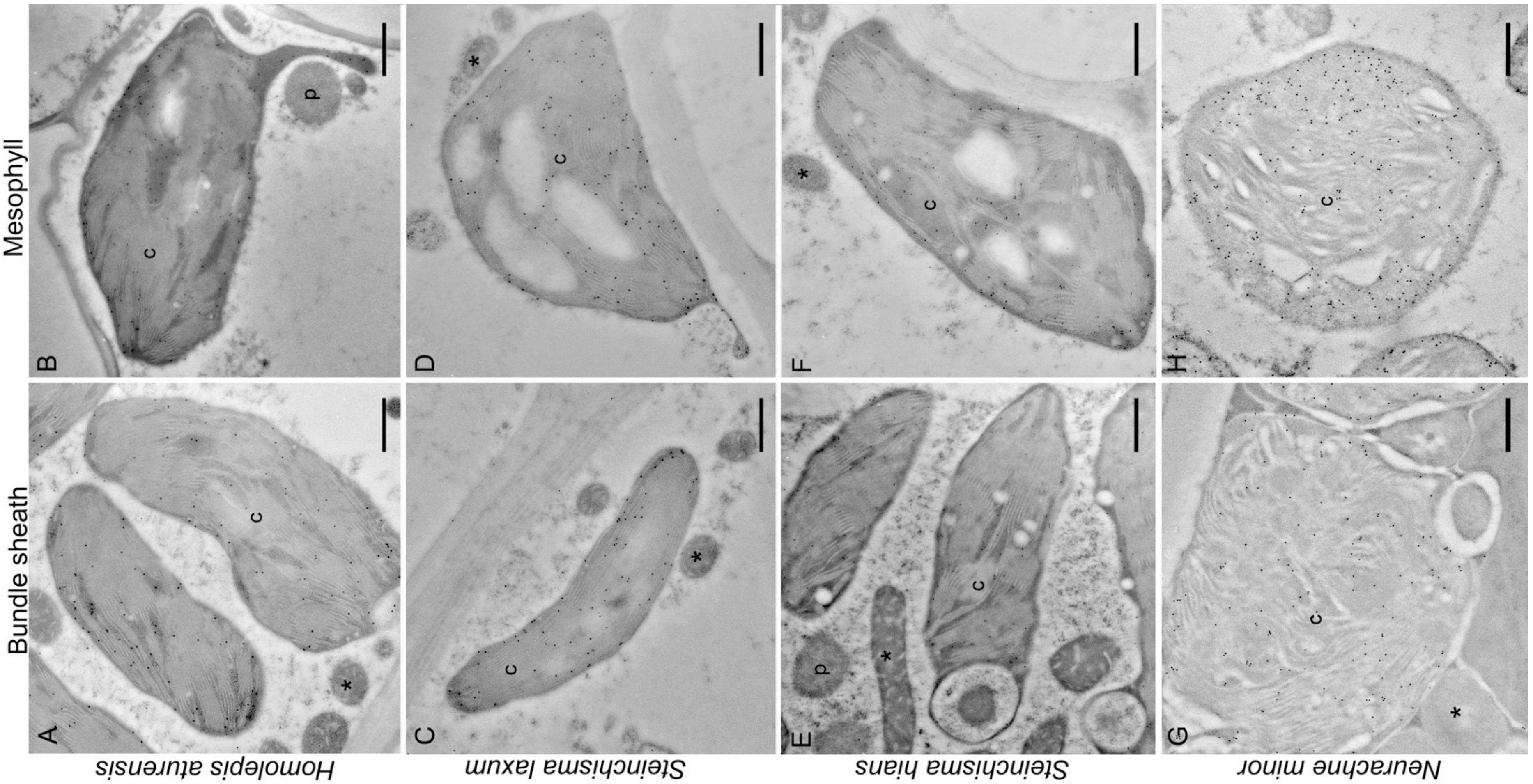

Supplementary Figure S6. Immunogold labeling for Rubisco large subunit in the bundle sheath and mesophyll cells of (A-B) *Dicanthelium oligosanthes* (C_3); (C-D) *Panicum bisulcatum* (C_3), (E-F) *P. virgatum* (C_4) and (G-H) *Setaria viridis* (C_4). p, peroxisomes; c, chloroplasts; asterisk marks mitochondria. Scale bars = 500 nm.

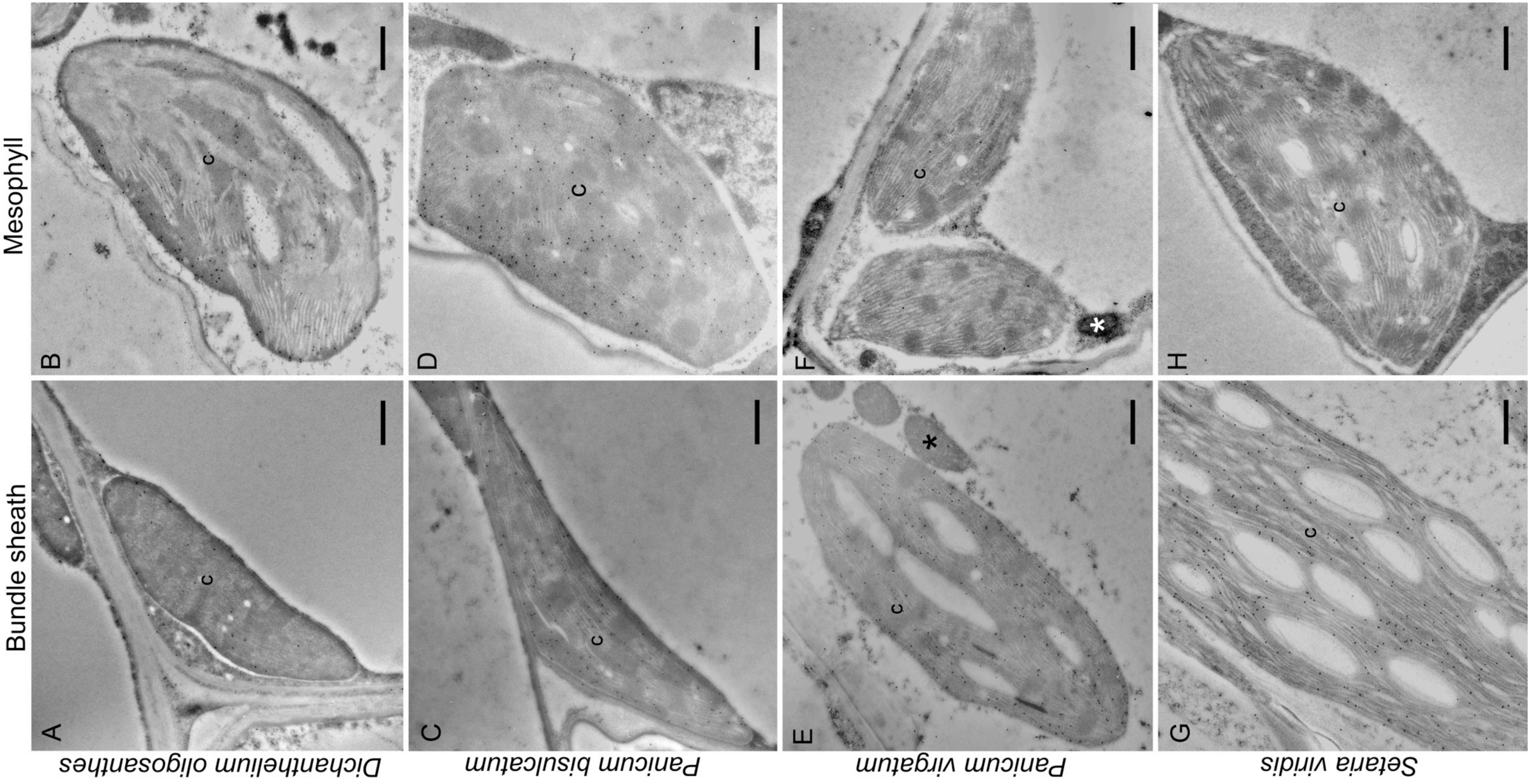

Supplementary Figure S7. A Bayesian phylogenetic tree of GLDH1, one of two GLDH paralogs conserved across all Angiosperms which includes the photorespiratory GLDH in *Arabidopsis* (AT2G35370). Dashed red lines denote inferred gene duplication events. Numbers at nodes indicate posterior probability.

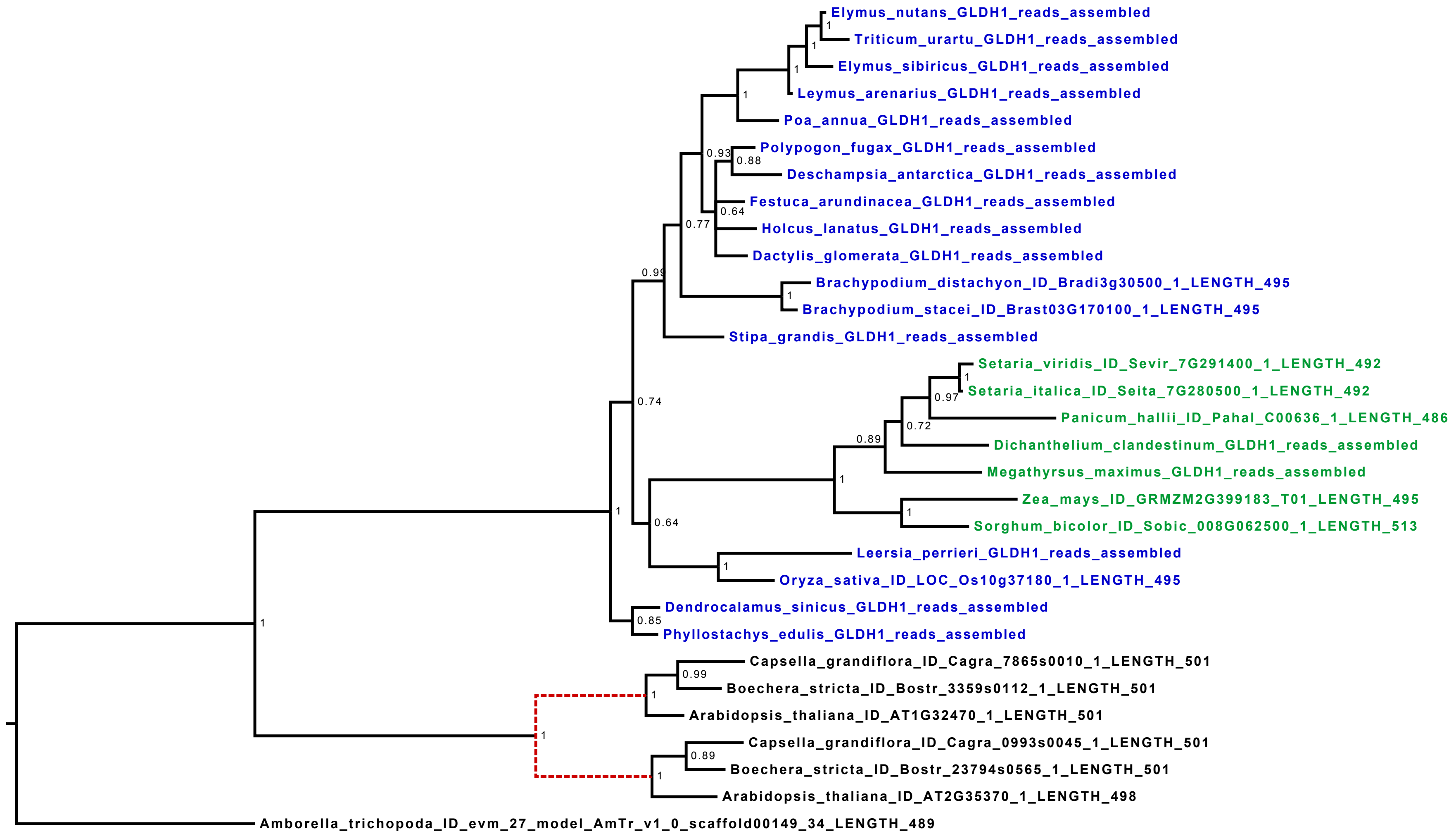

Supplementary Figure S8. A Bayesian phylogenetic tree of GLDH2, one of two GLDH paralogs conserved across all Angiosperms. Dashed red lines denote either gene duplication events or branches leading to duplicate copies. There is a duplication event in the Brassicaceae but *Arabidopsis* appears to have lost one copy. Numbers at nodes indicate posterior probability.

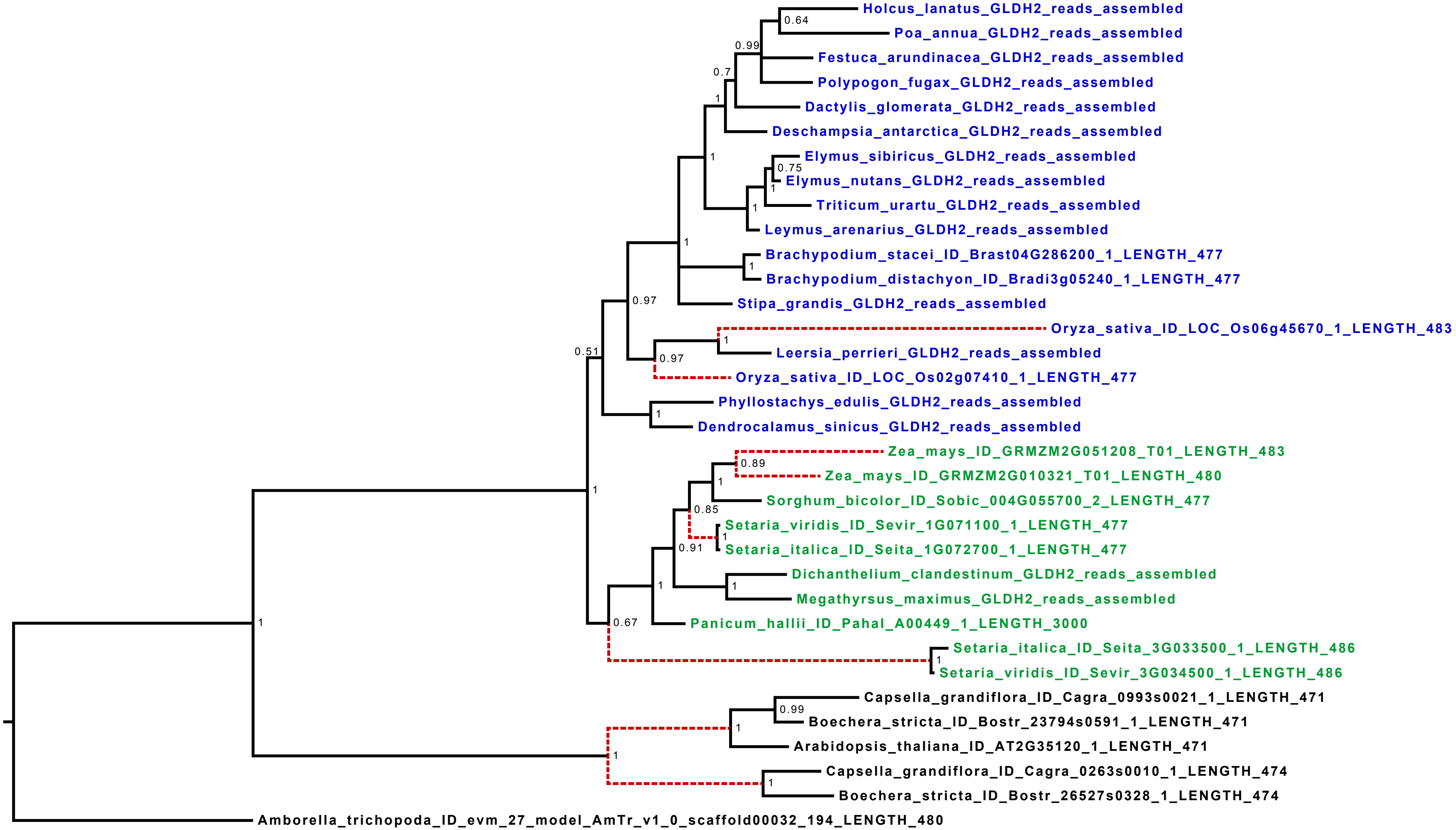

Supplementary Figure S9. A Bayesian phylogenetic tree of GLDL. Dashed red lines denote gene duplication events. This is the only GDC subunit with a grass-specific duplication. Most grass species examined possess both copies; however *Zea mays* and *Dendrocalamus sinicus* both appear to have lost GLDL2. Numbers at nodes indicate posterior probability.

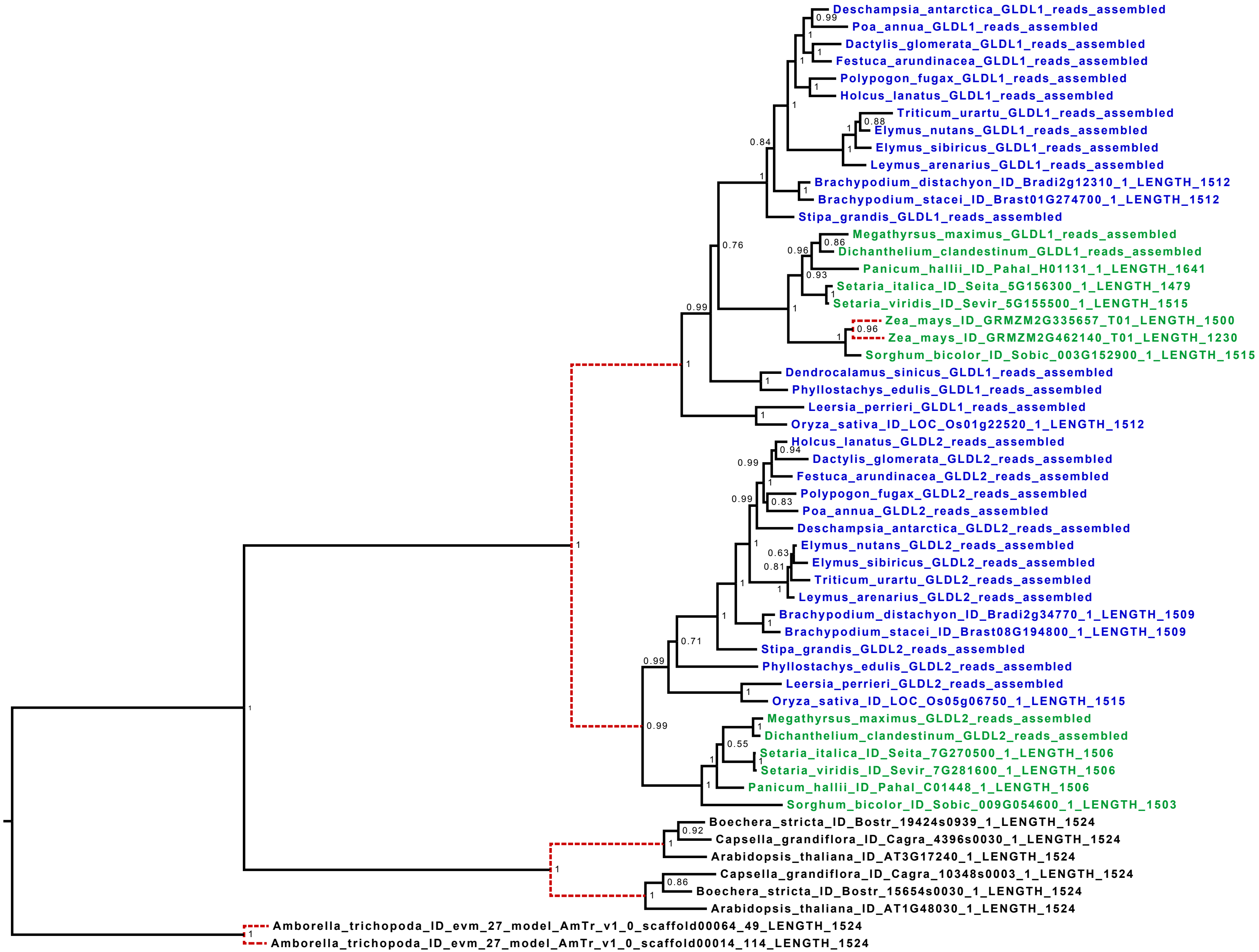

Supplementary Figure S10. A Bayesian phylogenetic tree of GLDT. No duplicates were found in any of the lineages examined. Numbers at nodes indicate posterior probability.

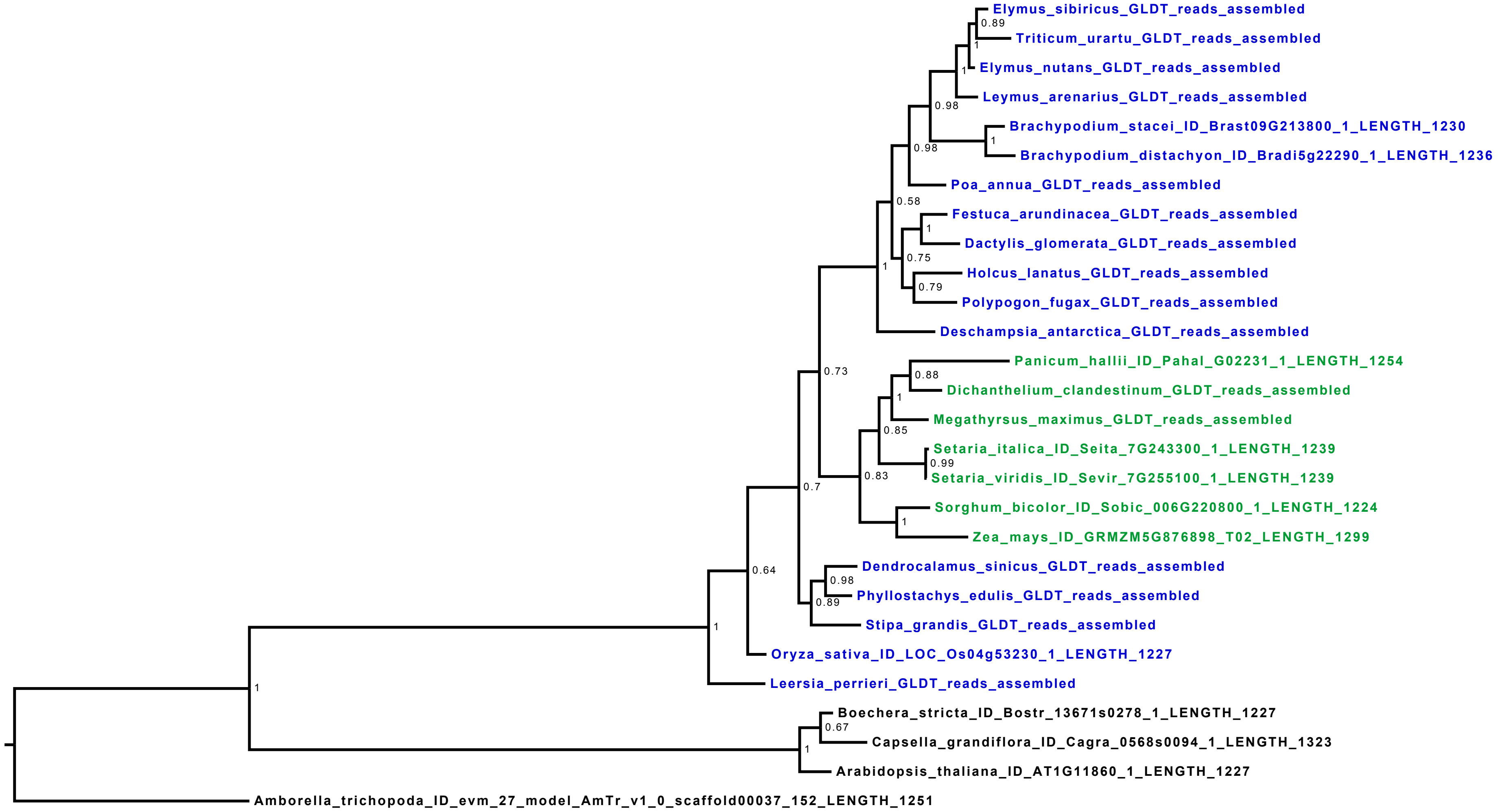

Supplement: Supplementary Data [file supp_erw150_supplementary_figures_S1_S10.pdf]
